# Supplementary material for: Limits of Predictability of Cascading Overload Failures in Spatially-Embedded Networks with Distributed Flows
Source: Sci Rep. 2017 Sep 15;7:11729. doi: 10.1038/s41598-017-11765-1 (PMC5601003; doi:10.1038/s41598-017-11765-1)
Supplement: Supplementary file 1 — Supplementary Information [file 41598_2017_11765_MOESM1_ESM.pdf]

Limits of Predictability of Cascading Overload Failures in Spatially-Embedded Networks with Distributed Flows
Supplementary Information

A. Moussawi[1,2], N. Derzsy[1,2], X. Lin[2,3], B. K. Szymanski[2,3], G. Korniss[1,2*]

August 8, 2017

1 Department of Physics, Applied Physics, and Astronomy, Rensselaer Polytechnic Institute, 110 8th Street, Troy, NY, 12180-3590 USA
2 Social Cognitive Networks Academic Research Center, Rensselaer Polytechnic Institute, 110 8th Street, Troy, NY, 12180-3590 USA
3 Department of Computer Science, Rensselaer Polytechnic Institute, 110 8th Street, Troy, NY, 12180-3590 USA

S.1 Distributed flow model

We assume that the flow is distributed, directed and of unit size, associated with a source and sink, and flow through all possible paths between source and sink. We model the network as a simple random resistor network with unit conductances along the edges [1, 2]. In this model each node and edge is involved in transporting current from source to sink, therefore each link experiences a load which is the current along that edge. For a link connecting nodes i and j the load is calculated as $\ell_{ij} = I_{ij}^{st}$, and the load on an arbitrary node i is the net current flowing through that node $\ell_i = I_i^{st}$. The two loads can be expressed as

$$I_i^{(st)} = \frac{1}{2}\sum_j |I_{ij}^{(st)}| \tag{1}$$

Next, we assume that all nodes are simultaneously sources and for each source we randomly choose a target from the remaning $N-1$ nodes. Thus, we assume that unit current flows simultaneously between N source/target pairs, and the load is defined as the superposition of all currents flowing through an arbitrary node. This is identical to the node current-flow betweenness[2, 3, 4, 5]:

$$\ell_{ij} = \frac{1}{N-1}\sum_{s,t=1}^{N} |I_{ij}^{(st)}|, \ell_i = \frac{1}{N-1}\sum_{s,t=1}^{N} |I_i^{(st)}|. \tag{2}$$

In order to obtain the I_{ij}^{st} currents along the edges from one source/target pair, we use Kirchhoff's law for each node i in the network and solve the system of linear equations:

$$\sum_{j=1}^{N} A_{ij}\left(V_i - V_j\right) = I\left(\delta_{is} - \delta_{it}\right), \forall i = 1, ..., N. \tag{3}$$

Here, we assume that I units of current flow through the network from source s to target t, and A_{ij} denotes the adjacency matrix of the network. This equation can be rewritten in terms of the weighted network Laplacian $\mathcal{L} = \delta_{ij}k_i - A_{ij}$, where $k_i = \sum_j A_{ij}$ is the degree of node i. Thus,

*E-mail: korniss@rpi.edu

we can write Eq. 3 as $\mathcal{L}V = \mathcal{I}$, where V is the unknown column voltage vector, and $\mathcal{I}_i$ is the net current flowing into the network at node i, and takes nonzero values only for the source and target nodes. Since the $\mathcal{L}$ network Laplacian is singular, we find the pseudo-inverse Laplacian $G = \mathcal{L}^{-1}$ using spectral decomposition [2, 6, 7]. Thus, by choosing as reference potential the mean voltage [6], $\hat{V}_i = V_i - \langle V \rangle$, where $\langle V \rangle = (1/N)\sum_{j=1}^{N} V_j$ for each node i we obtain:

$$\hat{V} = (GI)_i = \sum_{j=1}^{N} G_{ij} I \left(\delta_{js} - \delta_{jt}\right) = I \left(G_{is} - G_{it}\right) \tag{4}$$

Therefore, for I units of current and for a given source/target pair, the current flowing through edge (i, j) can be written as

$$I_{ij}^{(st)} = A_{ij} \left(V_i - V_j\right) = A_{ij} I \left(G_{is} - G_{it} - G_{js} + G_{jt}\right). \tag{5}$$

The above equation shows that current along an arbitrary edge is uniquely determined by network topology.

In modeling of the electrical flows in the power grid a commonly used approach is to use the DC power flow model [8, 9, 10, 11, 12], where links, in addition to resistance, also possess reactance. However, it has been shown in [8] that the equations for this DC model of power flow bear a close resemblance to that of an analogous electrical circuit. In prior studies it has also been demonstrated that, despite neglecting the true AC nature of the power grid, inferences made by employing the DC power flow model can still be useful[10].

It is important to point out that our goal is to study the fundamental aspects of cascades on spatial networks carrying ditributed flow, not designing strategies specifically tailored for electrical power transmission systems.

S.2 UCTE network properties

The Union for the Co-ordination of Transmission of Electricity (UCTE) data set [13] represents the power grid system of continental Europe during 2002. The network comprises $N = 1254$ transmission stations and $E = 1812$ edges spanning 18 European countries. The network is disassortative with an assortativity coefficient of 0.1, with average degree $\langle k \rangle = 2.889$ and clustering coefficient of $C = 0.127$. We model the system as a random resistor network carrying distributed flow and employ a capacity-limited overload model [14] to simulate cascading failures in the network (see Methods). Note that despite the simplicity of this fundamental model for conserved flows (i.e., Kirchhoff's and Ohm's law in the resistor network), the underlying system of equations have *identical structure* to those of the DC power-flow approximation (the current and voltage corresponding to the power and phase, respectively) [15, 16, 17].

We show in Fig. S1 that the load is positively correlated with the degree, while the degree and load distributions span a relatively narrow range, yet a significant variance of loads can be observed even for small degree values. This characteristic suggests that the load bearing responsibility of a node cannot be assessed exclusively from its degree. Using the spatial information of the nodes and edges, we plot the link length distribution (Suppl. Info. S2), and find that the majority of links span short distances with very few long range links constructed as part of the overall power grid designs. For the remainder of this article we use removal of a node, and attack on a node interchangeably to denote a node whose failure is used to initiate the cascade.

We define as the sensitivity of a node the size of the surviving giant component given that node fails in the system. In Fig. S2 we visualize the sensitivity of each node in the UCTE network. Red nodes indicate highly sensitive node, their individual removal leading to severe system damage.

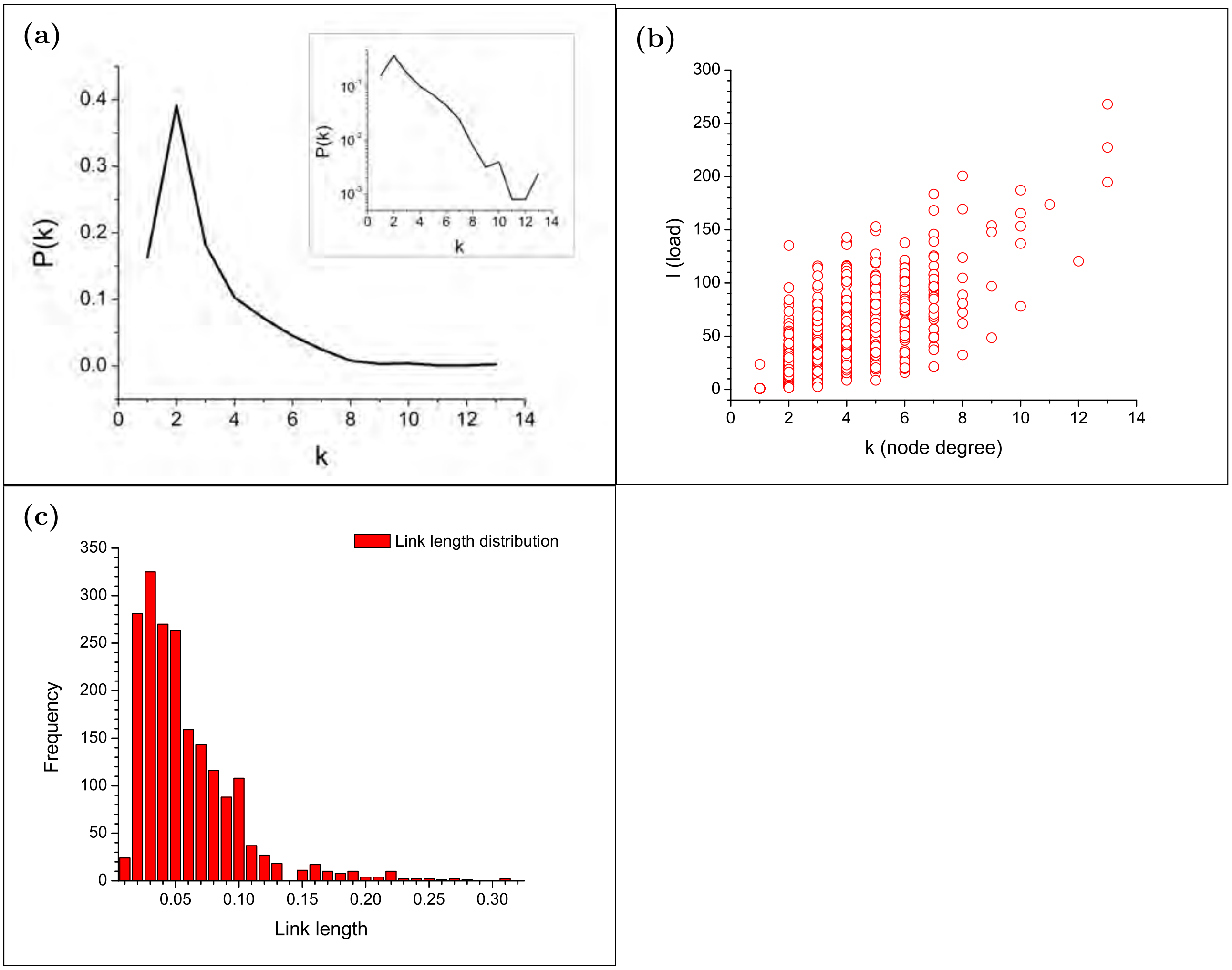

Figure S1: Properties of the UCTE network. (a) Node degree distribution; (b) correlation between node degree and node load; (c) histogram of link length distribution.

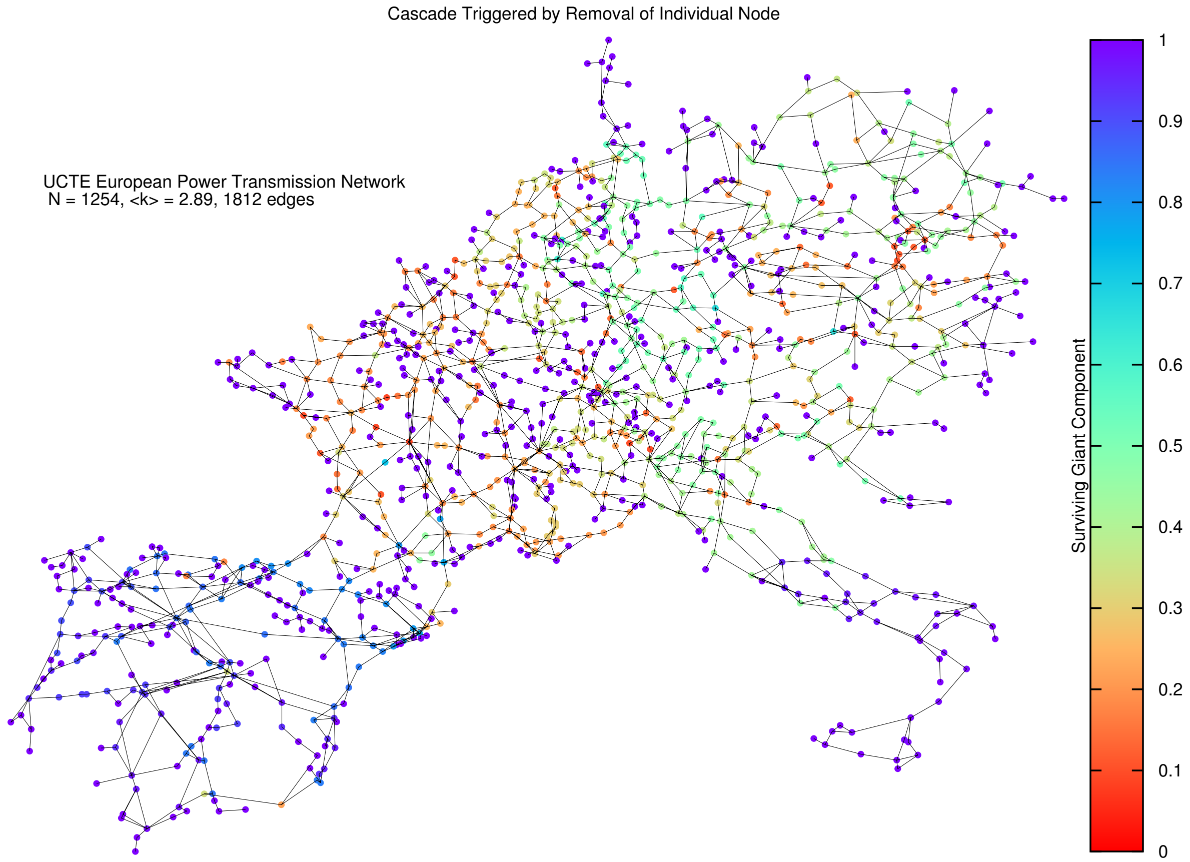

Figure S2: Node sensitivity of single-node removal in the UCTE network. The color hue indicates the size of damage (surviving giant component) in case that single node fails.

S.3 Random capacity assignment from heterogeneous distribution

The random capacity assignment from heterogeneous distributions have shown that we can stochastically find allocations that mitigate the cascading failures, but also can obtain realizations that lead to higher damage in the system than the uniform capacity allocation. Aiming to analyze the characteristics of excess capacity allocations, and to see whether we can observe if there are particular nodes that having higher capacity improve or diminish the protection of the system, we analyze in Fig. S5-**??** the correlation between node properties and excess capacity assignment for "best/worst" scenarios. We show that there is no apparent difference between these allocations that would explain the difference in the result.

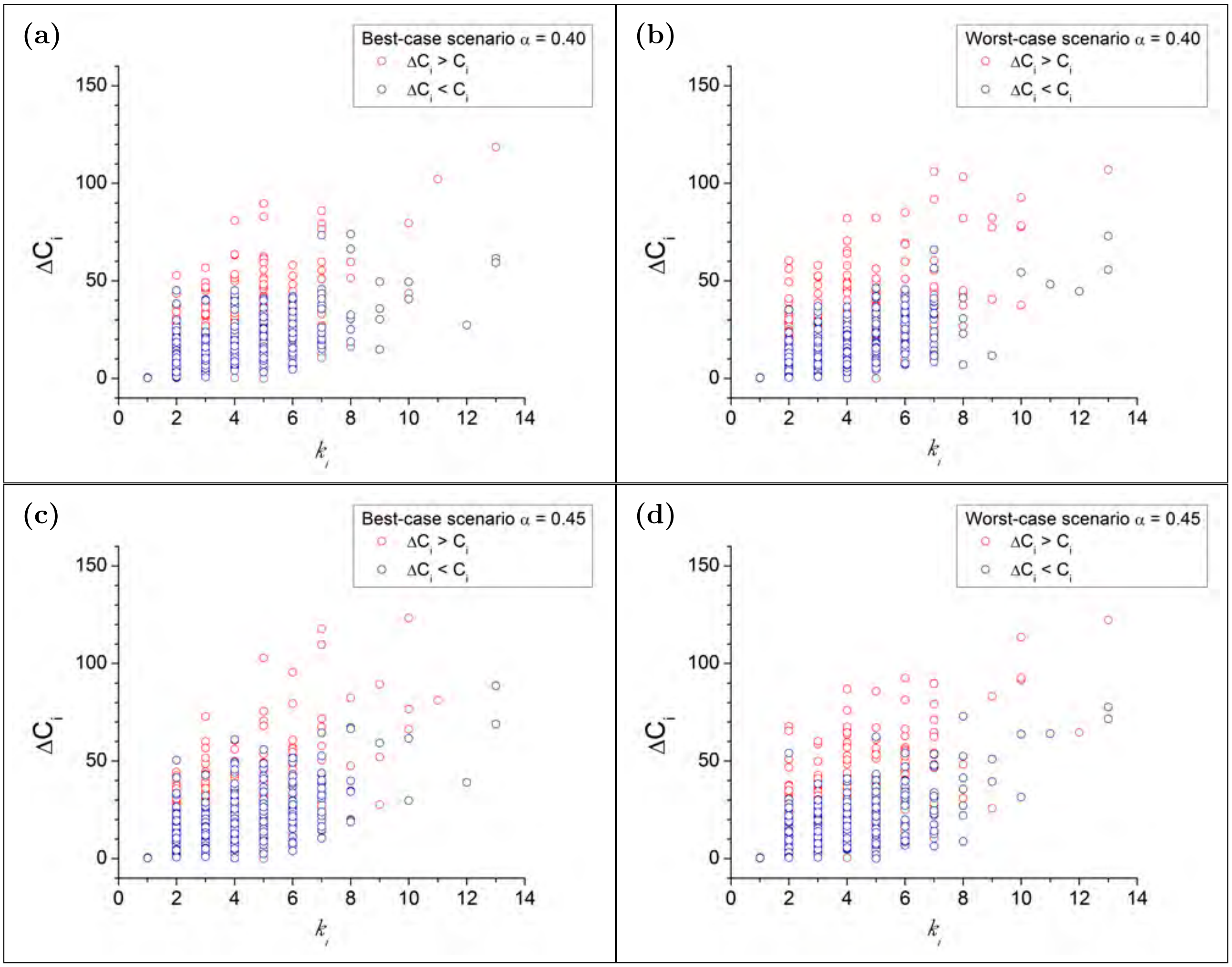

Figure S3: Comparison of best-case and worst-case excess capacity allocations as a function of node degree in the UCTE network. (a) Best-case for $\alpha = 0.40$, (b) worst-case for $\alpha = 0.40$, (c) best-case for $\alpha = 0.45$, (d) worst-case for $\alpha = 0.45$.

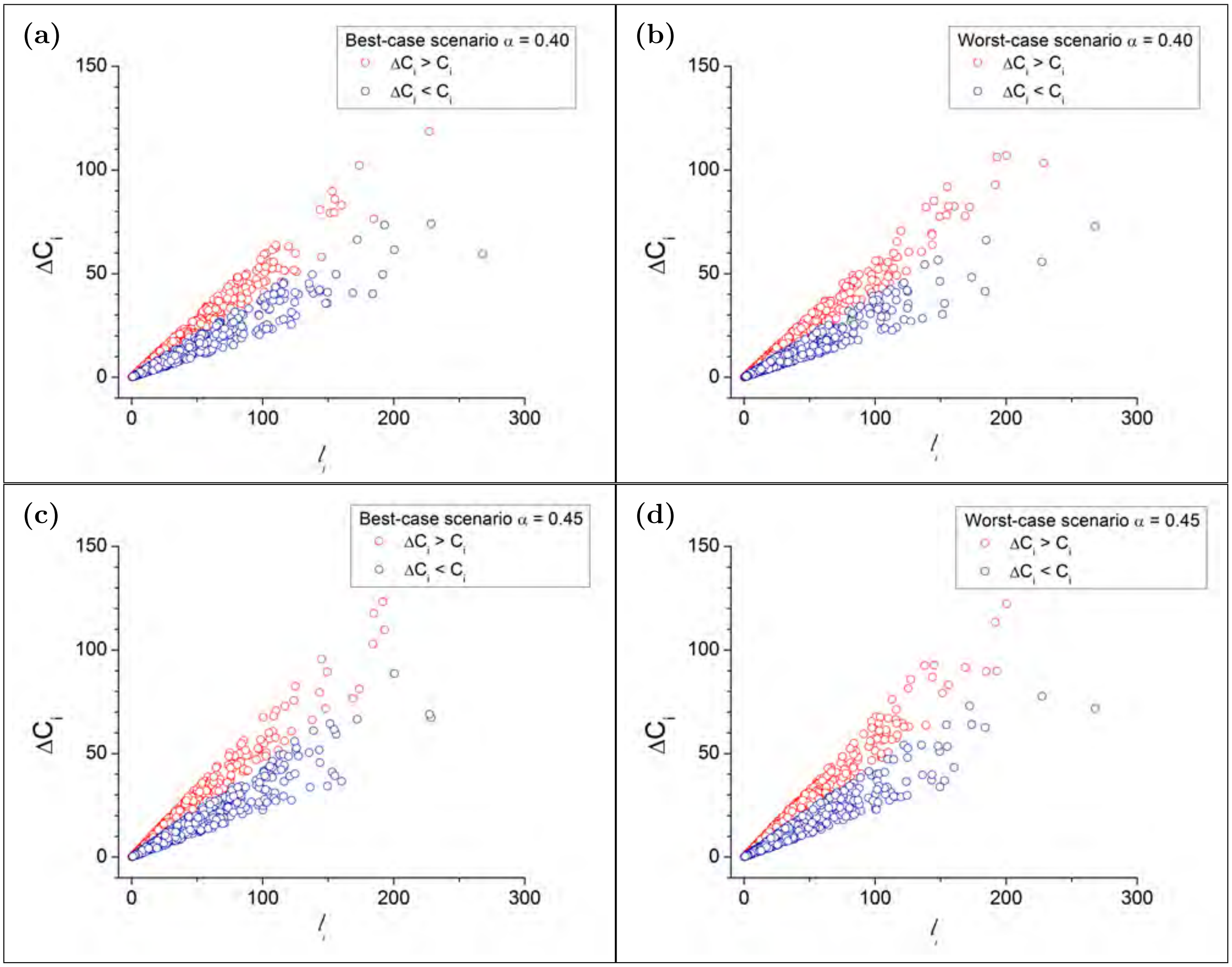

Figure S4: Comparison of best-case and worst-case excess capacity allocations as a function of initial state node load in the UCTE network. (a) Best-case for $\alpha = 0.40$, (b) worst-case for $\alpha = 0.40$, (c) best-case for $\alpha = 0.45$, (d) worst-case for $\alpha = 0.45$.

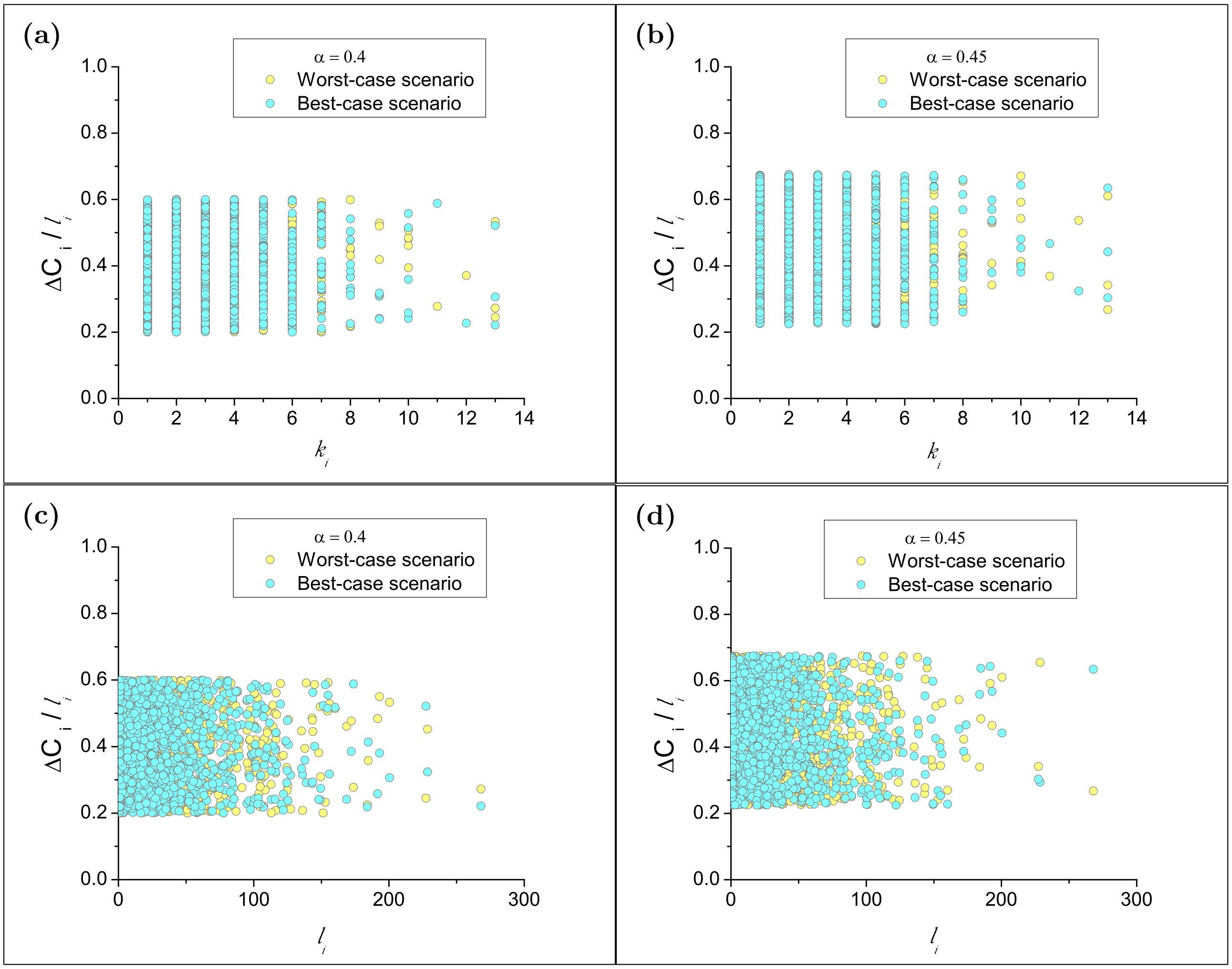

Figure S5: Comparison of best-case and worst-case excess capacity allocations in the UCTE network. (a) Comparison of excess capacity, normalized by initial state load, as a function of node degree for $\alpha = 0.40$ and (b) $\alpha = 0.45$. (c) Comparison of excess capacity, normalized by initial state load, as a function of initial state node load for $\alpha = 0.40$ and (d) $\alpha = 0.45$.

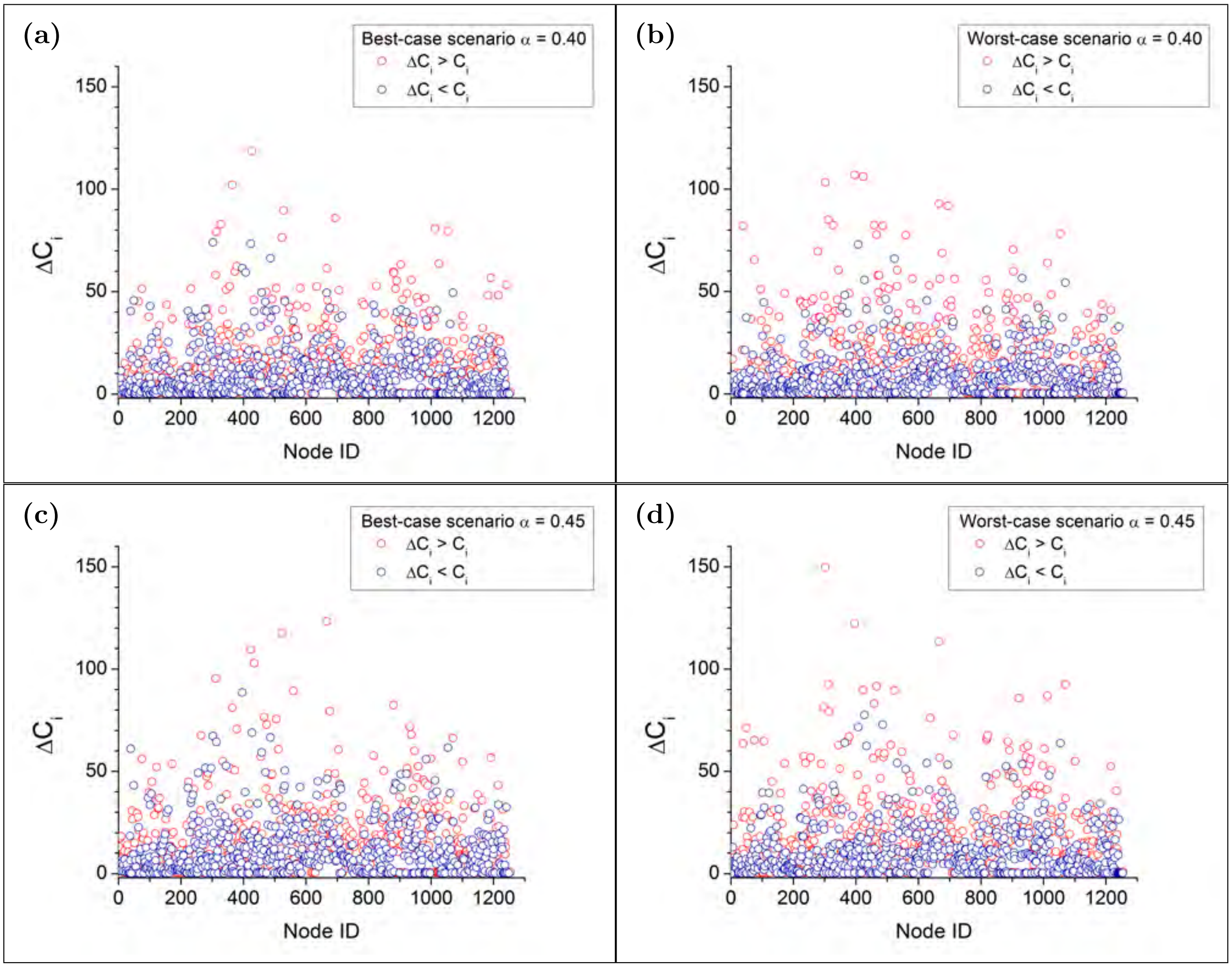

Figure S6: Comparison of best-case and worst-case excess capacity allocations by node ID in the UCTE network. (a) Best-case for $\alpha = 0.40$, (b) worst-case for $\alpha = 0.40$, (c) best-case for $\alpha = 0.45$, (d) worst-case for $\alpha = 0.45$.

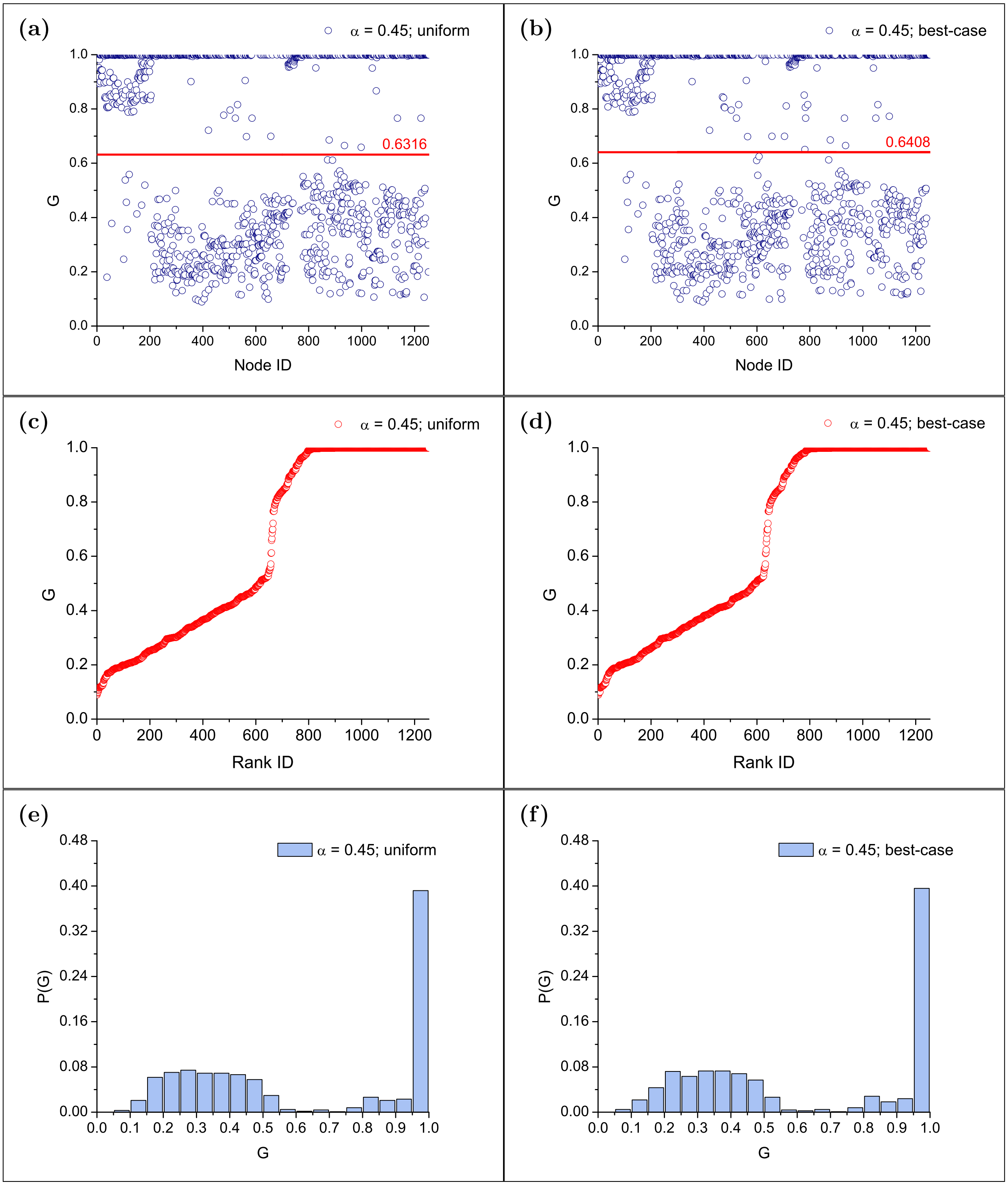

Figure S7: Comparison of UCTE network with uniform capacity allocation (left) and UCTE network with stochastic best-case capacity allocation (right). (a)-(b) Node sensitivity for single-node failure. The red line highlights the averaged value of the data points. (c)-(d) Node sensitivity for single-node failure rank ordered by the size of surviving giant component. (e)-(f) Histograms visualizing the distribution of the size of surviving giant component for single-node failures.

S.4 Non-monotonic behavior in spatially-correlated attacks in the UCTE network vs the tolerance.

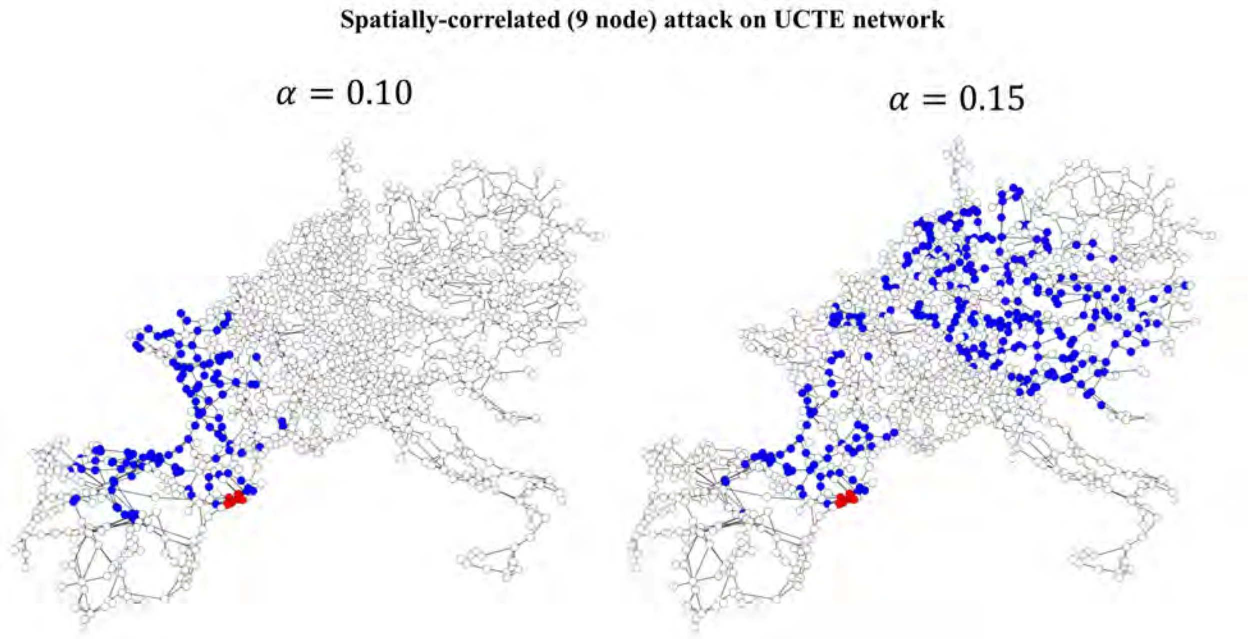

Figure S8: Non-monotonic behavior in spatially-correlated attacks in the UCTE network vs the tolerance. We trigger the cascades by removing the same set of 9 nodes (red) from the $1r$ region in the UCTE network for two different tolerance (α) values. We show that a smaller tolerance parameter leads to a smaller damage (left), whereas a higher tolerance parameter of $\alpha = 0.15$ (right) causes a larger cascading failure (see Movie S12-13). This result suggests the presence of "fuse" nodes, which can prevent the spreading of cascades in case they fail due to a lower capacity allocation.

S.5 Sensitivity to Target Selection in Spatially-Localized Multi-Node Attacks

S.5.1 Node sensitivity in RGGs and Scale-Free Networks

So far we have studied the node sensitivity of the UCTE network. Next, we generate artificial networks to analyze and compare the sensitivity of nodes in different network constructions. We find in Fig. S9 that random geometric graphs (RGG), similarly to UCTE system show no correlation between sensitivity and node degree, whereas scale-free (SF) networks exhibit a correlation between node degree and sensitivity. Similar behavior can be found in low $\langle k \rangle = 3$ average degree as well as high $\langle k \rangle = 10$ average degree RGG and SF systems.

We analyze the severity of cascading failures in RGG networks of various system sizes and varying α values in Fig. S10.

S.6 *N*-Stable and *(N-1)*-Stable Capacity Allocation for the UCTE network

In Fig. S12 we analyze the required capacity allocations for each node in the case of N-stable UCTE in comparison with the *(N-1)*-stable scenario.

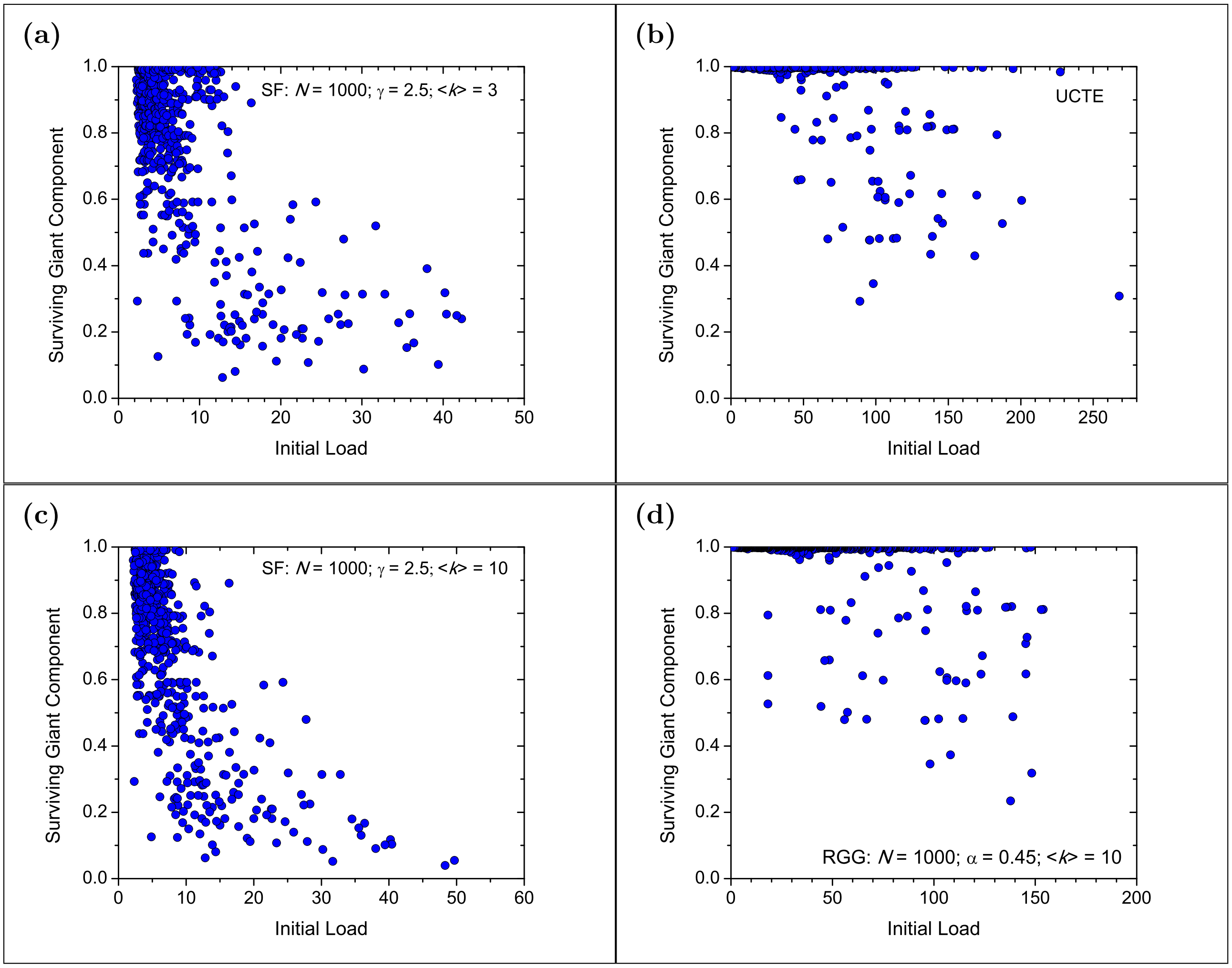

Figure S9: Node sensitivity analysis for single-node removal. The subfigures plot the size of the surviving giant component (in case the cascade is triggered by the removal of a single-node) as a function of node load in (a) scale-free (SF) network with system size $N = 1000$ and $\langle k \rangle = 3$, (b) UCTE network, (c) scale-free (SF) network with system size $N = 1000$ and $\langle k \rangle = 10$; (d) RGG with system size $N = 1000$ and $\langle k \rangle = 10$. Each network is constructed with $\alpha = 0.45$ tolerance parameter.

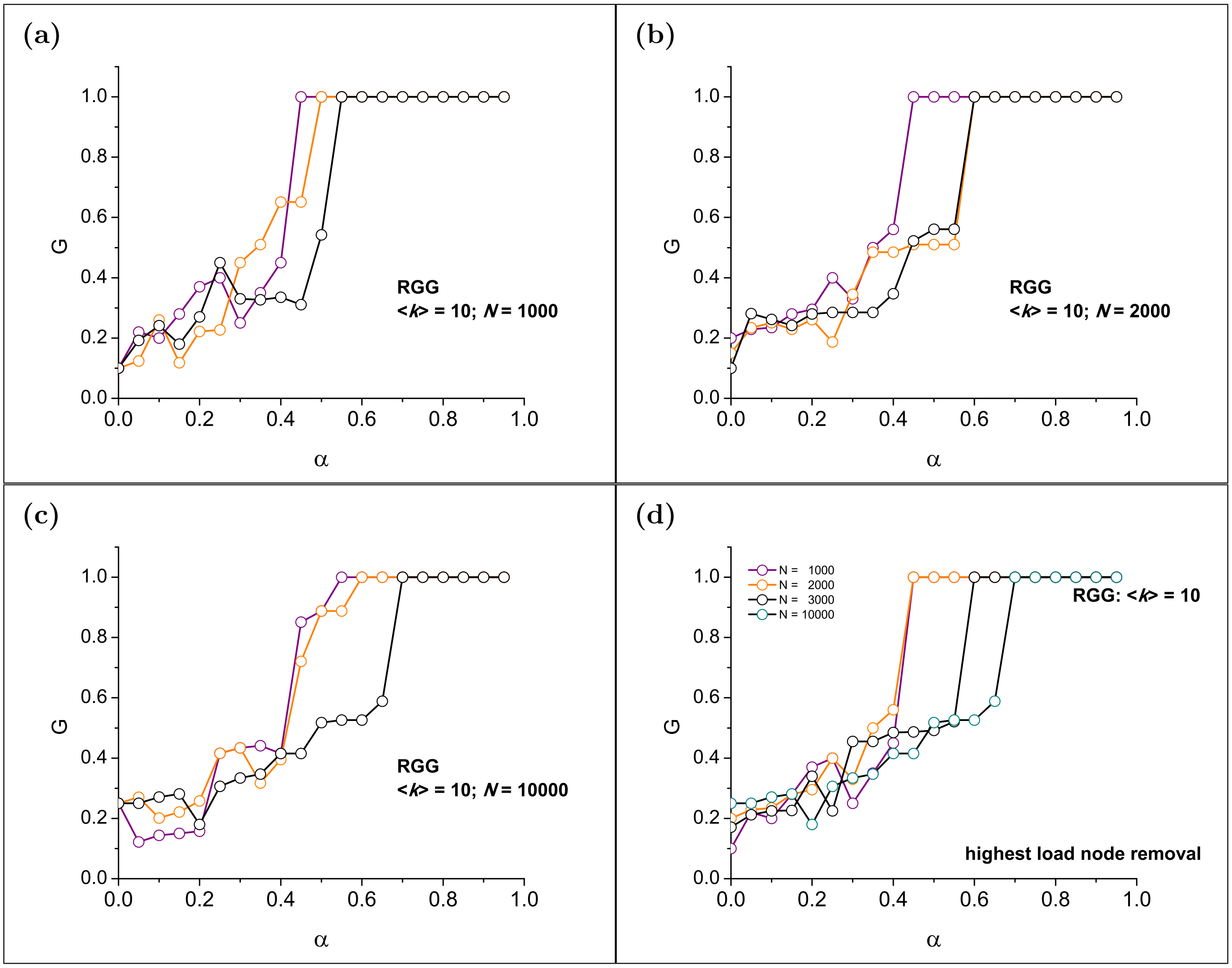

Figure S10: Sample-to-sample fluctuation and finite-size behavior of cascading failures on RGGs. Cascades triggered by the removal of the highest load node on RGG networks of size (a) $N = 1000$, (b) $N = 2000$, (c) $N = 10000$, with 3 different realizations for each system size. (d) Analysis of non-monotonic behavior scaling with system size. Simulations performed on synthetic random geometric graphs (RGGs) of average degree $\langle k \rangle = 10$ and various N system sizes.

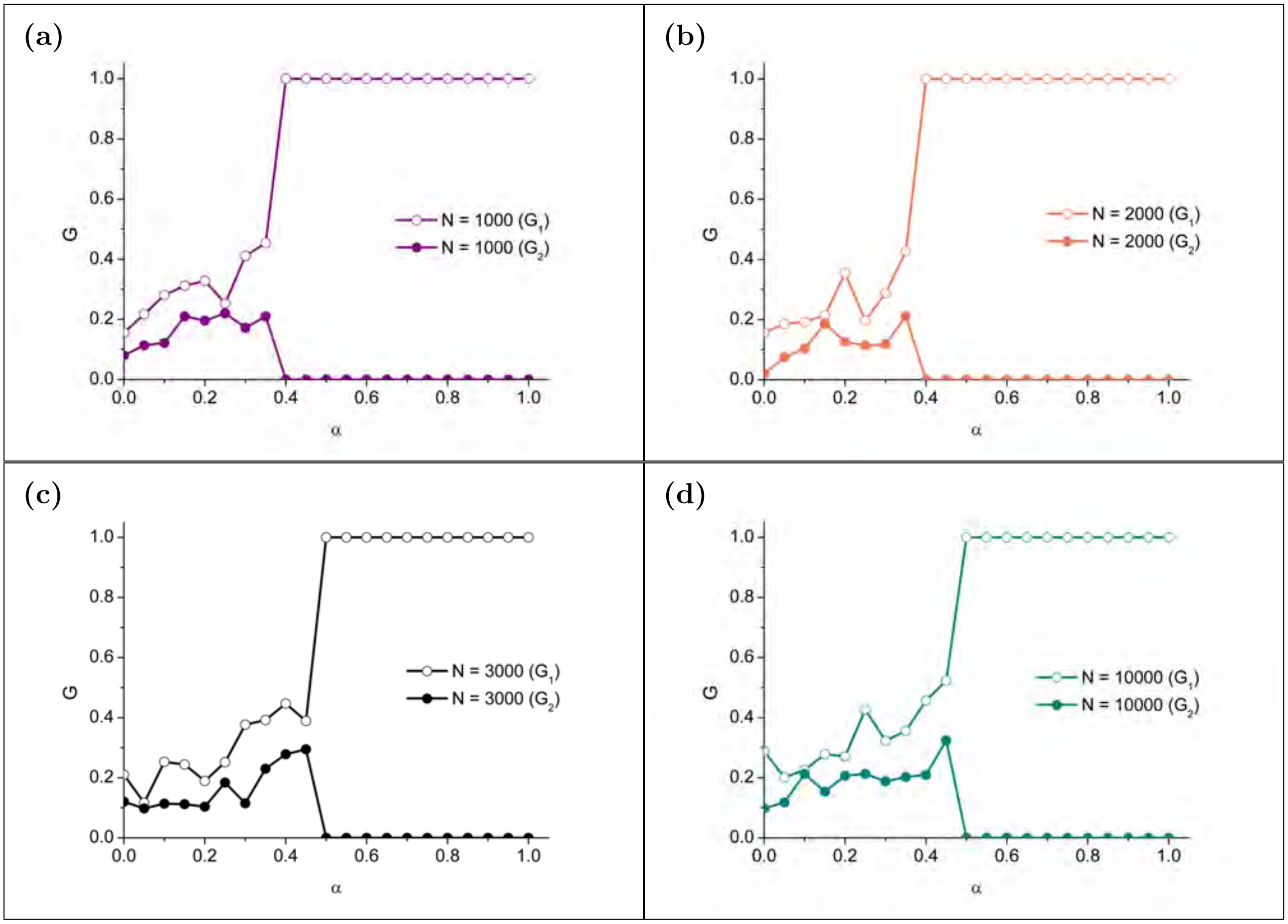

Figure S11: Comparison of the largest surviving giant component (G_1) and second largest giant component (G_2s) in RGGs. Cascades triggered by the removal of the highest load node on RGG networks of size (a) $N = 1000$, (b) $N = 2000$, (c) $N = 3000$, (d) $N = 10000$ and average degree $\langle k \rangle = 10$.

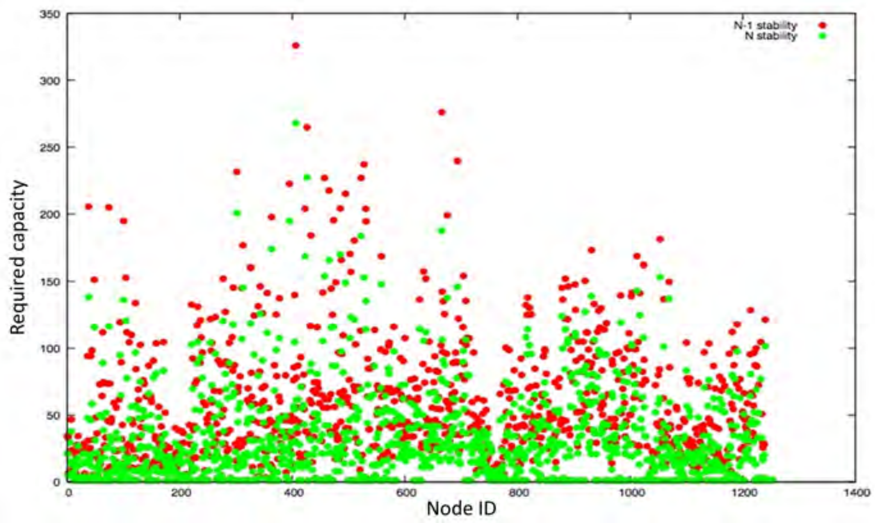

Figure S12: Required capacity comparison for N-stable and *(N-1)*-stable UCTE network constructions.

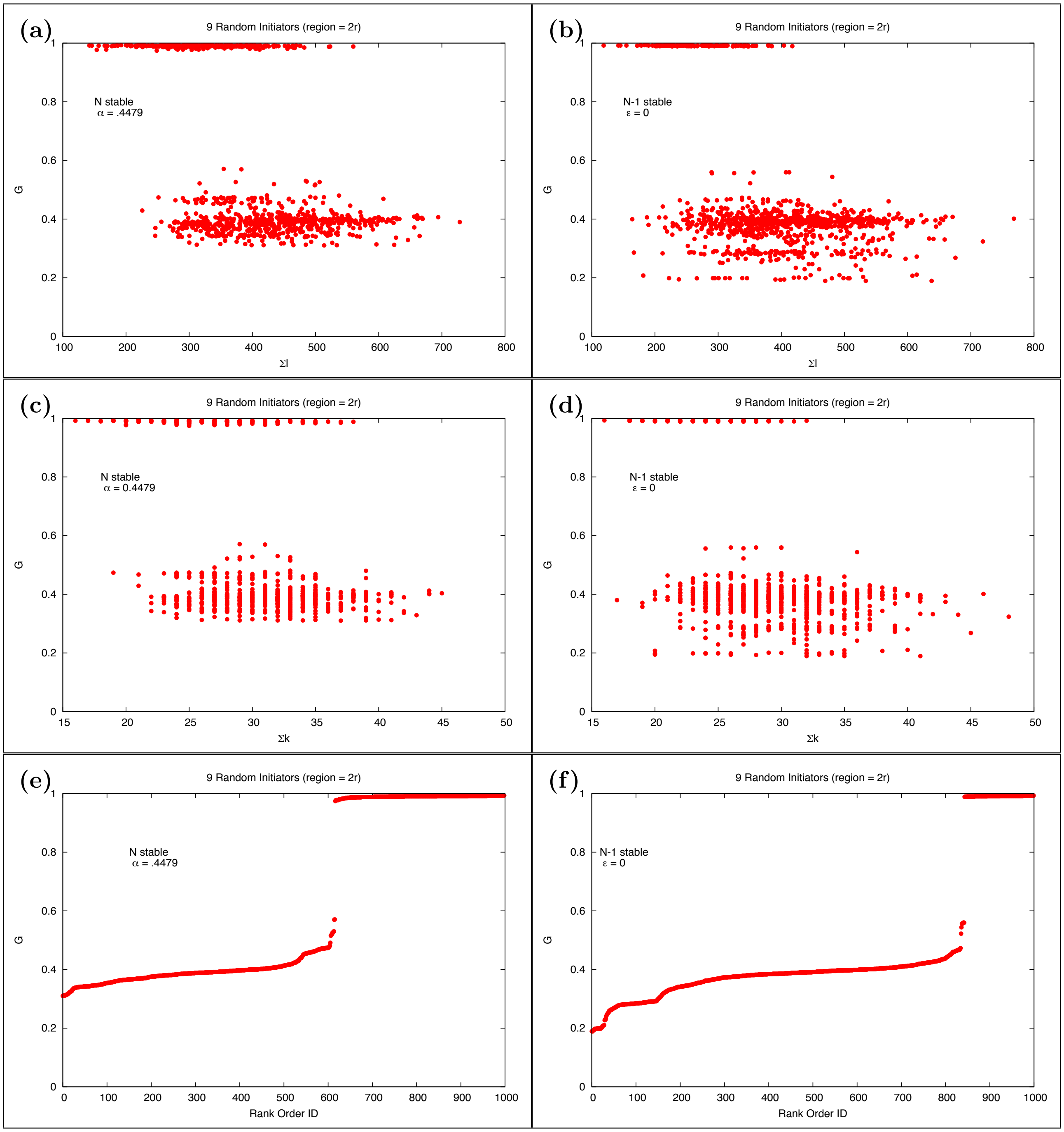

Figure S13: Nine-random-node removals from $2r$ region for (a) N-stable and (b) *(N-1)*-stable UCTE network.

in Fig. S14 analyze the cascading failures in the N-stable and *(N-1)*-stable UCTE networks by removing multiple nodes, and identify "fuse" nodes, the nodes that when survive transport the load to regions which lead to higher damage.

S.6.1 Stochastic Capacity Allocation

In Fig. S15 we study the efficacy of stochastic capacity allocations for the N-stable UCTE network constructions. We stochastically search for the best capacity allocation that provides the highest protection to the network in case of nine-center-node attacks in the N-stable network. Next, we use the best allocation to assign these excess capacities to the systems, and analyze its robustness in case of nine-random-node failures in the entire network. We find that the stochastic optimization

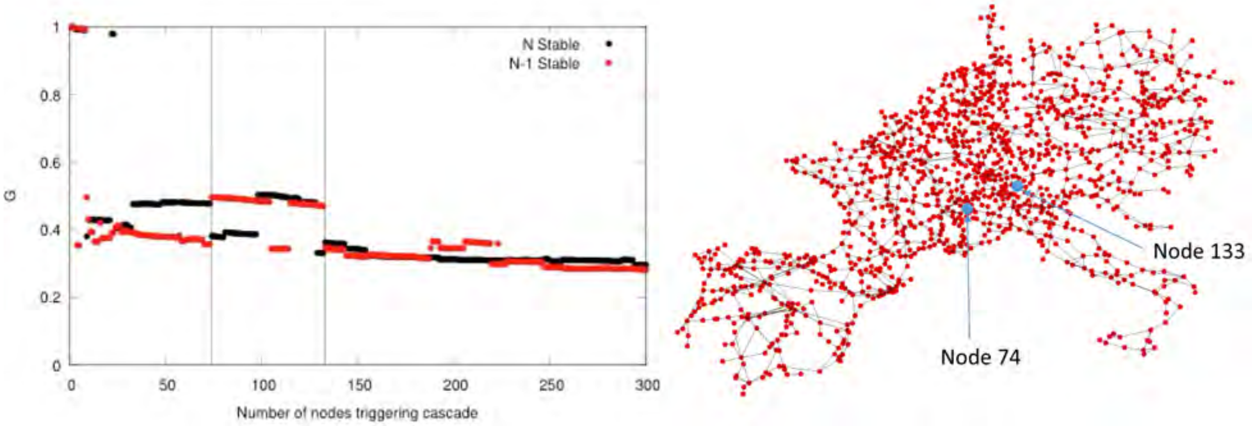

Figure S14: Stability of N-stable and *(N-1)*-stable UCTE networks against multi-node attacks. The two blue nodes on the UCTE network visualization depict critical nodes (fuses) that increase the damage in the system in case they survive.

allocation makes the network more vulnerable to random attacks/failures. This implies that by increasing the protection of the systems against a known attack strategy, we reduce the stability of the system against unaccounted attacks.

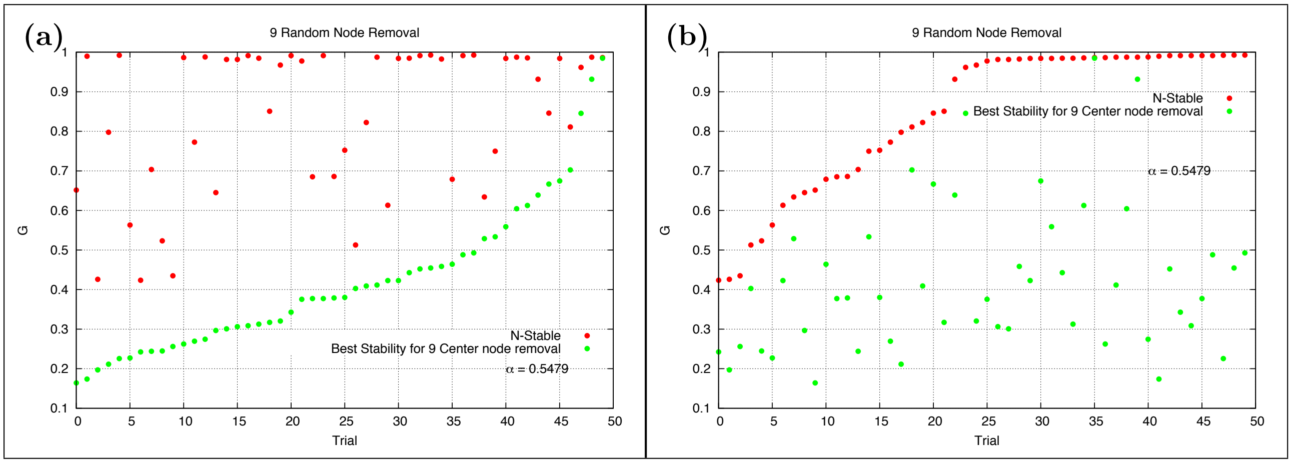

Figure S15: Stochastic capacity allocation optimization for N-stable UCTE network. The red data points depict the stability of the (a) N-stable UCTE networks against nine-random-node removal from the entire network. (b) Trials ordered by G. The green data points highlight the vulnerability of the network, if we add on top of the initial capacity allocation the best stochastic capacity allocation against nine-center-node removals.

S.7 Multi-node Attack Strategies

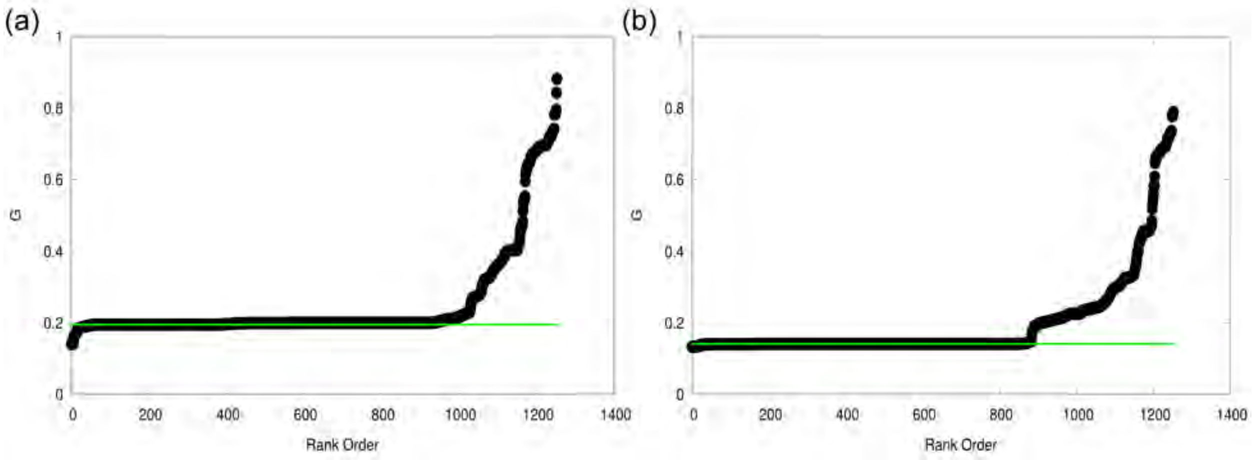

Figure S16: Greedy selection of (a) 2 most damaging nodes with every other node; (b) 3 most damaging nodes with every other nodes. The green lines represent the size of surviving giant component in case of the removal of the (a) 2 most damaging nodes only; (b) the 3 most damaging nodes only. Simulations performed on N-stable UCTE network with α=0.5927.

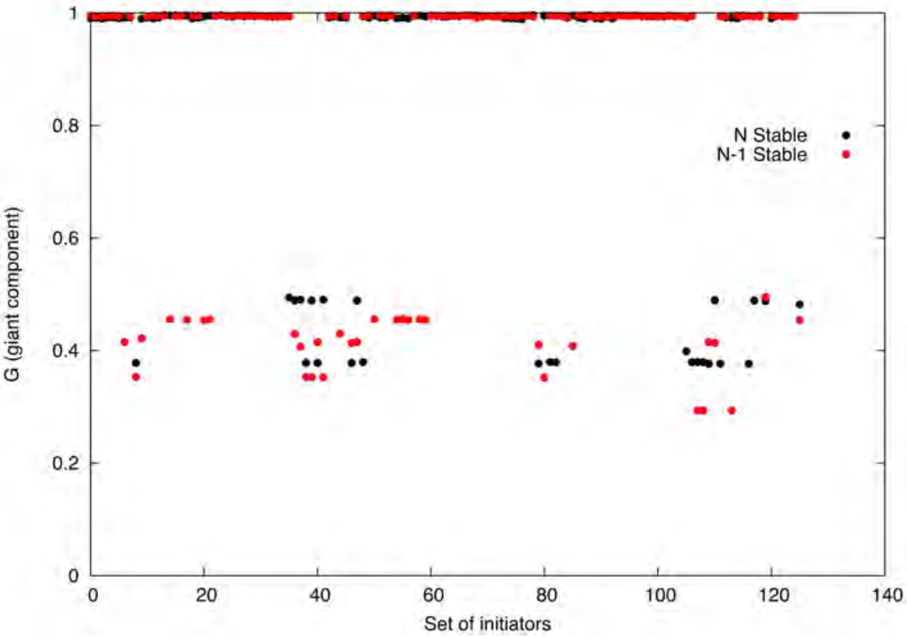

Figure S17: Five-center-node removal. From the nine-center-nodes we attack different 5 nodes for each simulation, leading to 126 possible combinations. Simulations performed on N-stable UCTE network (black) with α=0.5927 and *(N-1)*-stable UCTE network (red) with ϵ=0.1.

S.8 Phase Transition in Cascading Failures

We analyze the power-law trend of the cascade size distributions of the UCTE network in three regions: for α values below the phase transition, around the phase transition and above the phase transition. We find that around the phase transition region the cascade size distributions exhibit clear power-law behavior. However, as we move away from the phase transition region (both above and below), we can see the power-law trend vanish. In comparison, we have performed the same analysis on RGG networks, and found similar characteristics.

S.9 Cascade-size Distribution Analysis

S.9.1 Cascade-size Distribution using G and S

We show that there is a strong linear correlation between G (surviving giant component) and S (number of failed nodes), thus we can use these two measures interchangably to quantify the severity of a cascade.

S.9.2 G_1 vs G_2

G_1 is the size of the largest surviving giant component, whereas G_2 is the size of the second largest giant component. We study the correlation between their sizes and their values vs. α. We study the G_1 and G_2 correlations by removing four-center-nodes from the UCTE network, similarly to the RGG networks presented in Fig. S11.

S.9.3 Kolmogorov-Smirnov (KS) Test

In order to ensure the accuracy of our power-law fitting, we use the Kolmogorov–Smirnov test (K–S test or KS test) [18] to validate our results. In statistics, this method is a nonparametric test of the equality of continuous, one-dimensional probability distributions used to compare a

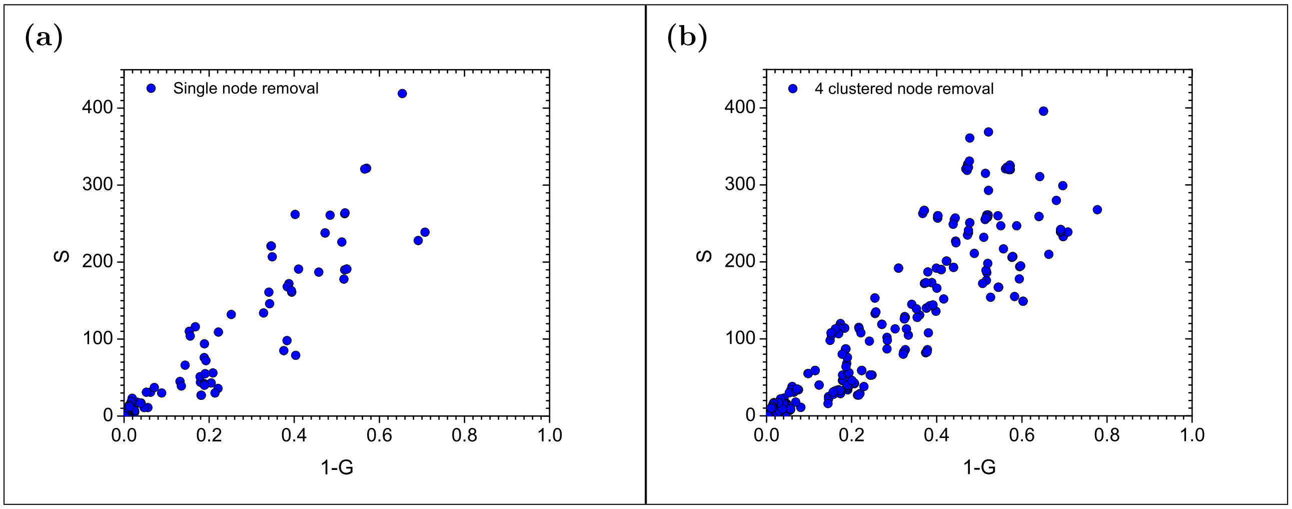

Figure S18: Correlation analysis of S vs. G. S represents the number of failed nodes throughout the cascade process, whereas G is the size of surviving giant component. In order to quantify the same information (size of damage), we plot the 1-G that is the fraction of failed nodes. (a) The severity of single-node removal; (b) severity of four-spatially-clustered-node removals. Simulations performed on N-stable UCTE network with $\alpha = 0.5927$.

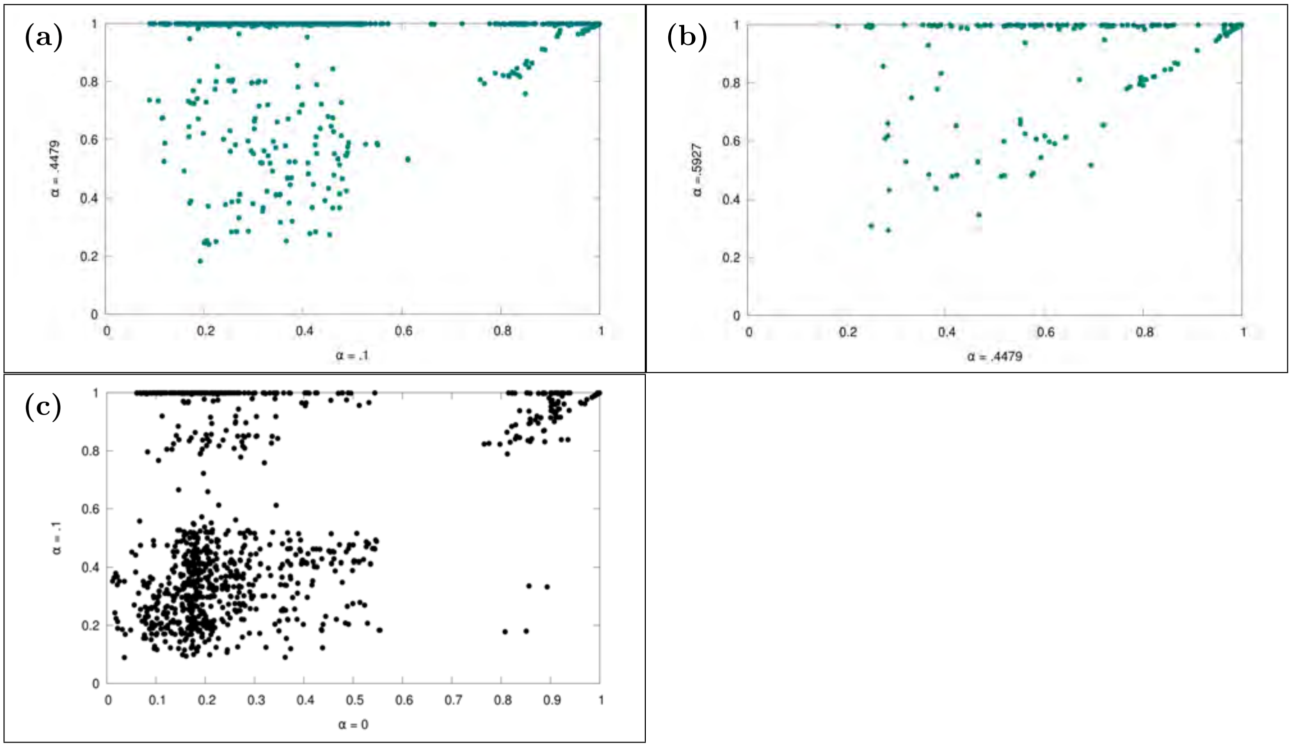

Figure S19: Correlation between surviving giant component sizes for all possible single-node attacks on the UCTE network with two N-stable capacity thresholds as represented on the axes.

sample with a reference probability distribution (one-sample K–S test), or to compare two samples (two-sample K–S test). As the authors point out, the least-squares method can give inaccurate estimates of parameters for power-law distribution, and in addition, they give no indication of whether the data does indeed follow a power law [19]. Therefore, based on this seminal work, we employ statistical testing using the maximum-likelihood fitting (MLE) to estimate the accuracy of the fitted power-law, and KS test for validating the goodness of fitting a power-law. Our empirical results are obtained as probability density function (PDF), denoted as $p(X)$ (see Suppl. Info. S8),

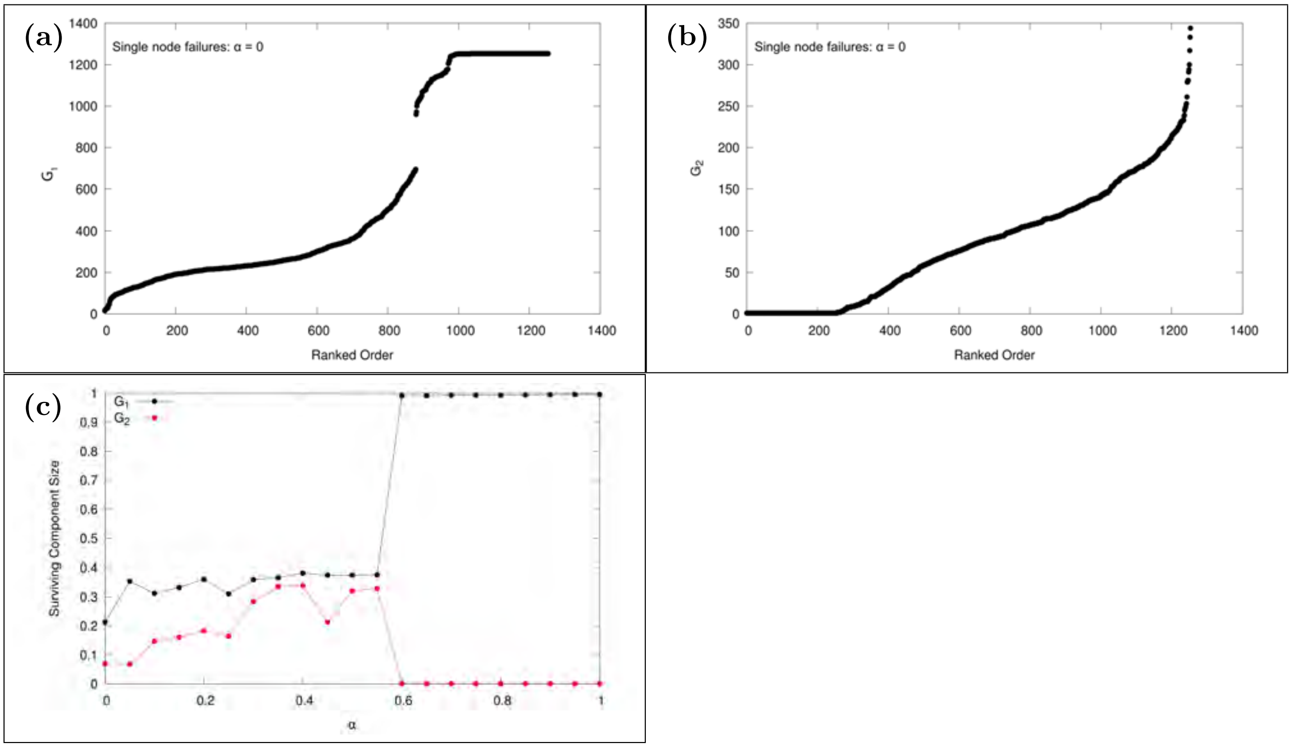

Figure S20: Comparison of surviving giant component and second largest surviving component. (a) The surviving giant component (G_1) in ranked order, (b) the second largest component (G_2) in ranked order, as each single-node in the UCTE network is individually attacked, with a capacity threshold $\alpha = 0$. (c) The surviving giant component size (G_1) and second largest component size (G_2) as a function of capacity threshold α under a four-center-node attack on the UCTE network. A phase transition is visible.

however the KS-statistic requires the cumulative distribution function (CDF), which we obtain as $P_>(S) = \sum_{X=S_{min}}^{S} p(X)$. Finally, we extract the slope by performing a least-squares linear regression on the logarithm of the cumulative distribution function.

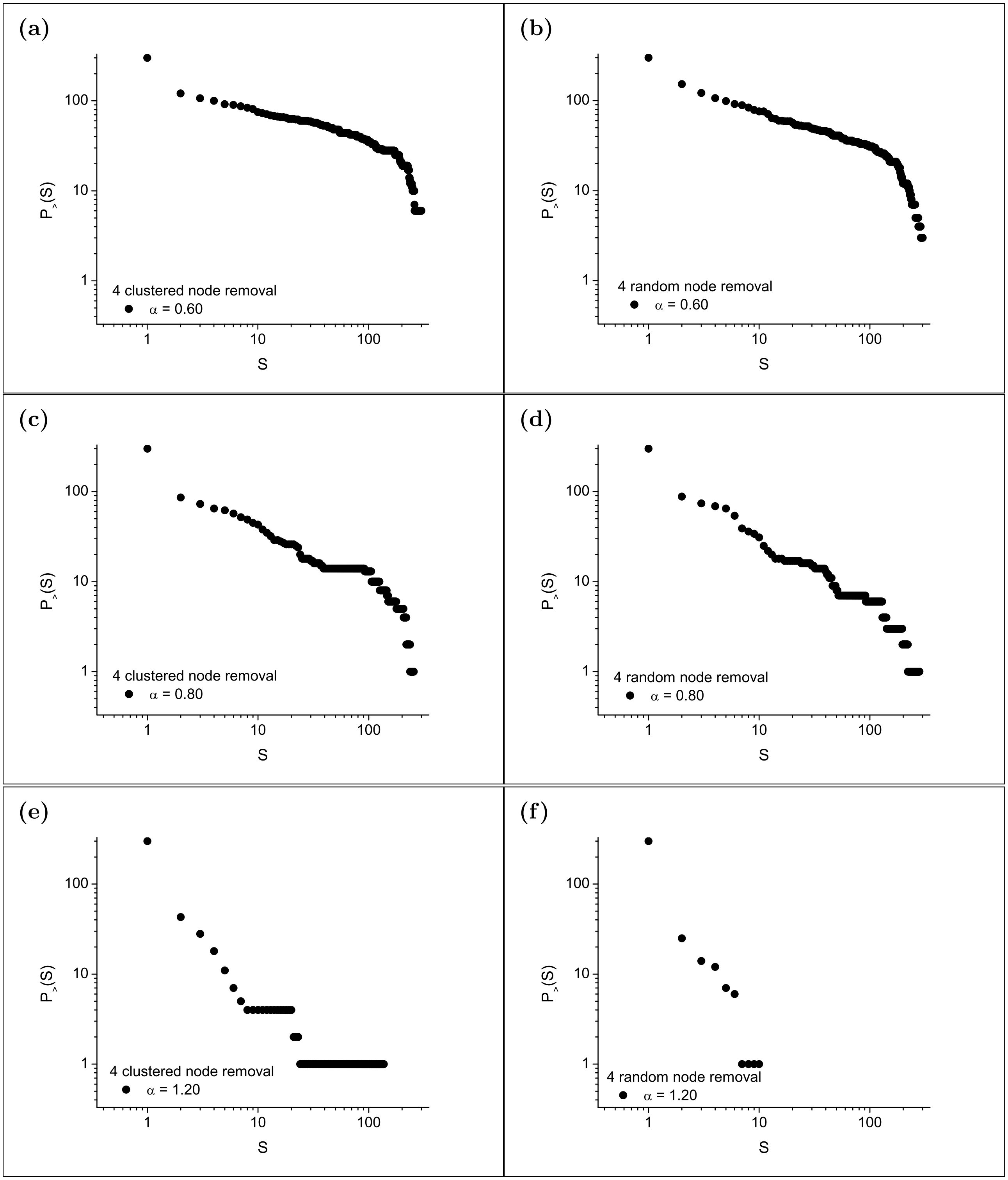

Figure S21: Power-law trend around the phase transition region in N-stable UCTE network. Comparison shown for four-random-node removal and four-clustered-node removal for various α values: below ($\alpha = 0.6$), around ($\alpha = 0.8$) and above ($\alpha = 1.2$) the phase transition region).

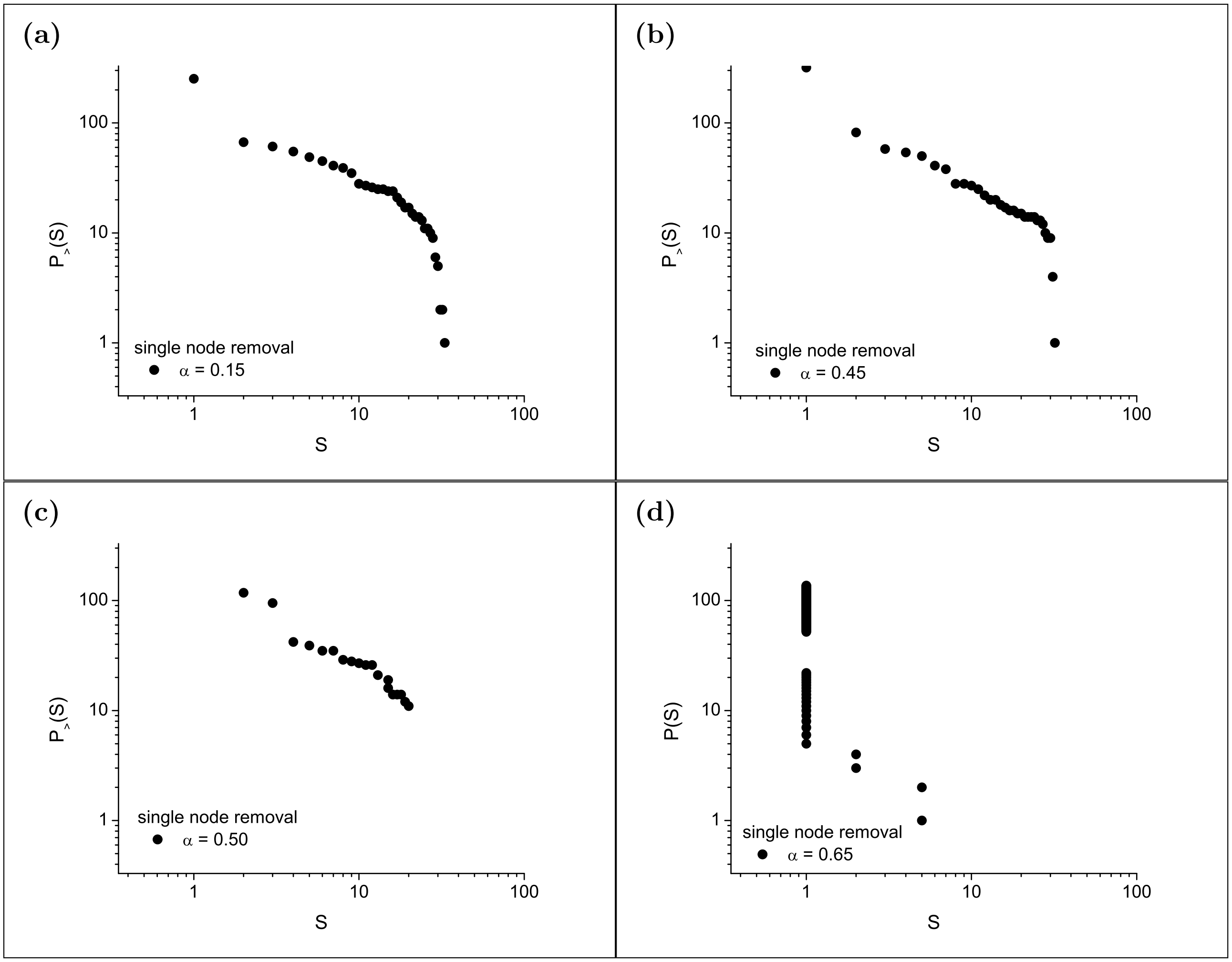

Figure S22: Power-law trend around the phase transition region in RGG network of size $N = 1000$ and $\langle k \rangle$. Cascades triggered by the removal of single-nodes for various α values: below ($\alpha = 0.15$), around ($\alpha = 0.45$ and $\alpha = 0.50$) and above ($\alpha = 0.65$) the phase transition region).

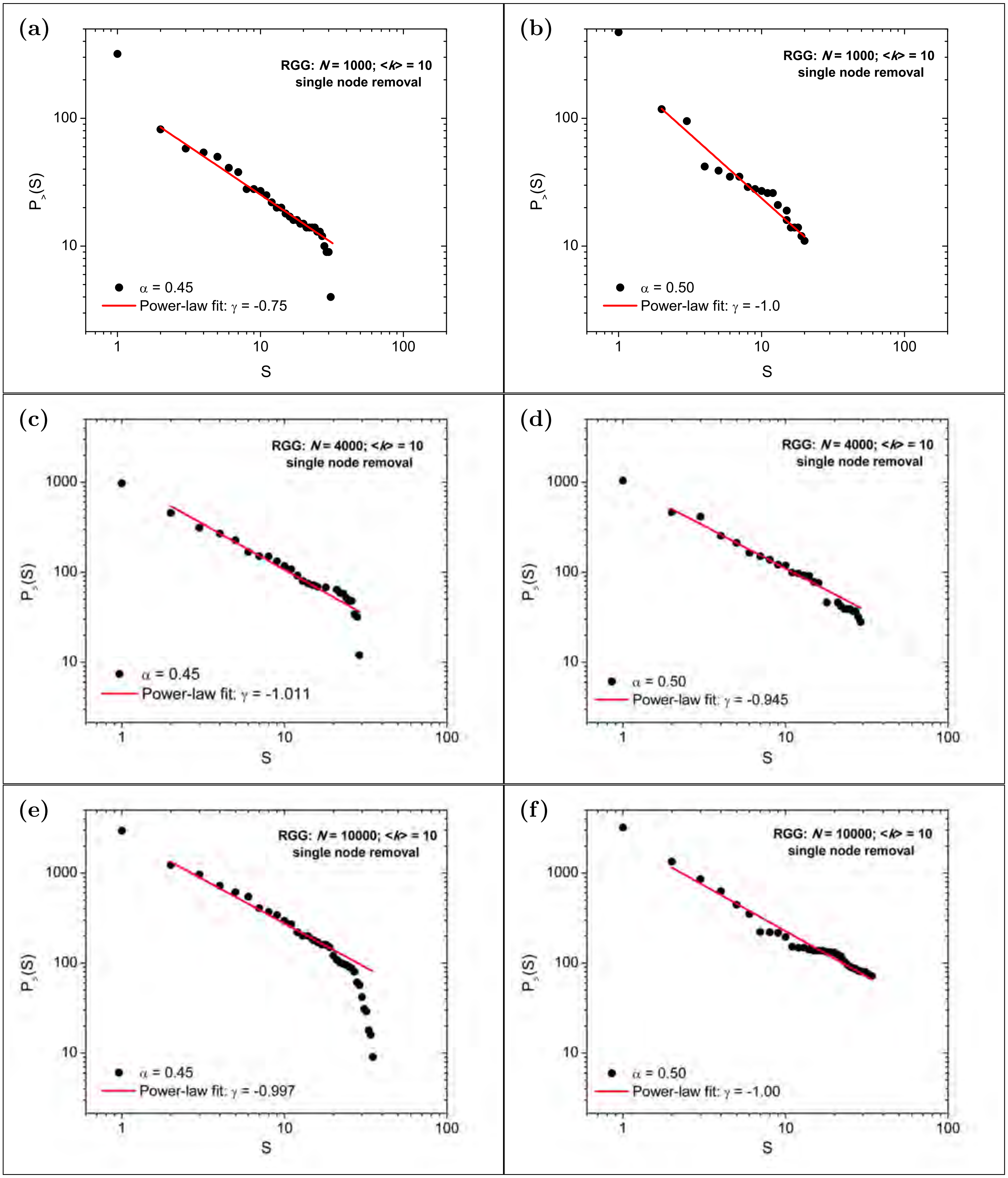

Figure S23: Power-law fits around the phase transition region in RGG networks of various system sizes and $\langle k \rangle = 10$. Cascades triggered by the removal of single-nodes for various α values: at the critical region ($\alpha = 0.45$) and beyond ($\alpha = 0.50$)

S.10 Predicting the Severity of Cascading Failures

Next, we analyze the predictability of cascading failures in the UCTE network triggered by multiple random node failures based on the knowledge of the severity of each cascading failure that each node causes individually. We show that when the number of triggering nodes (n) is small ($n \leq 4$) we can accurately predict the severity of induced damage. However, as the number of nodes that trigger the cascade increases, the predictability becomes ineffective.

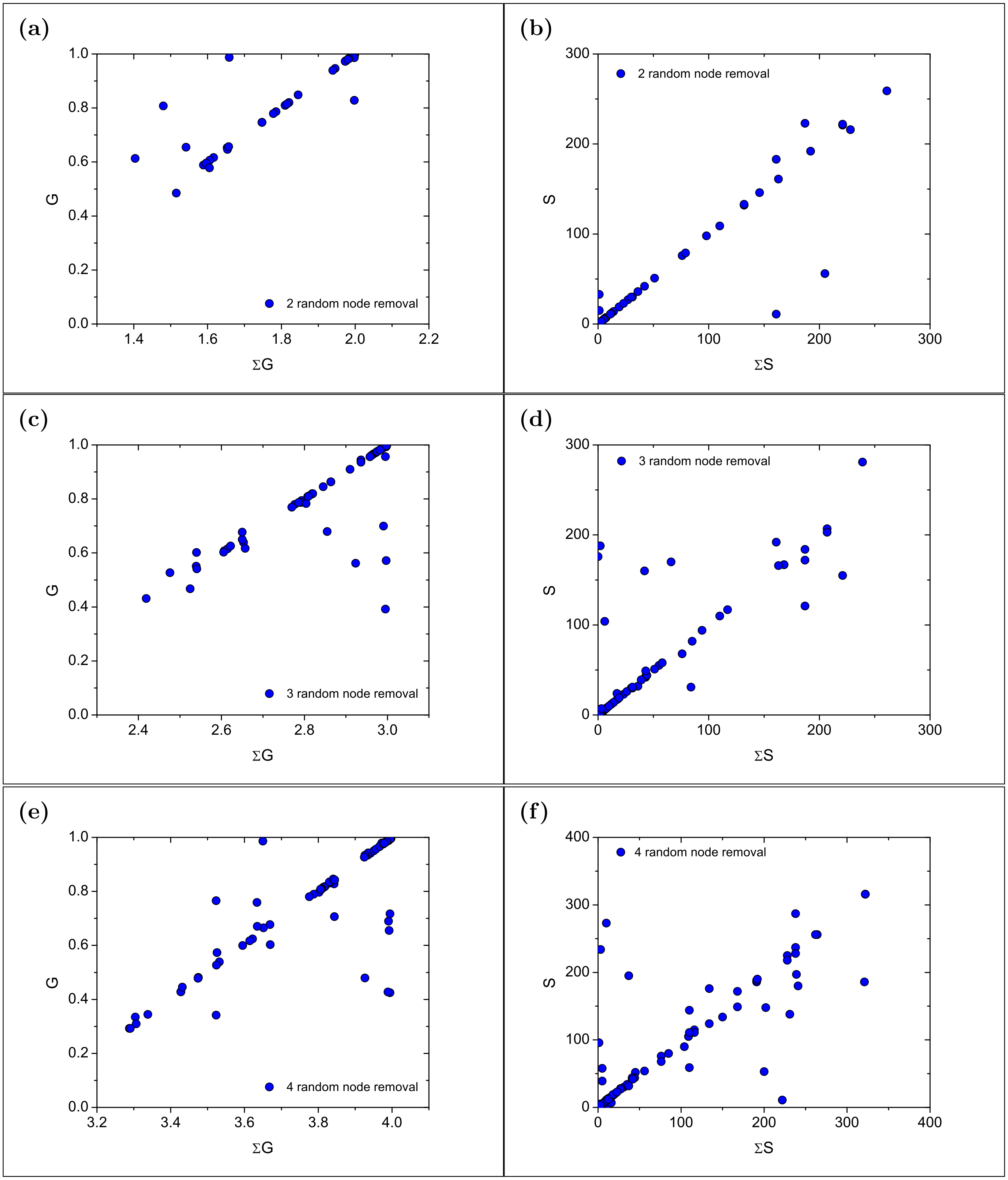

Figure S24: Correlation analysis of multi-node attacks and the sum of individual damage each initiator node induces in N-stable UCTE network.

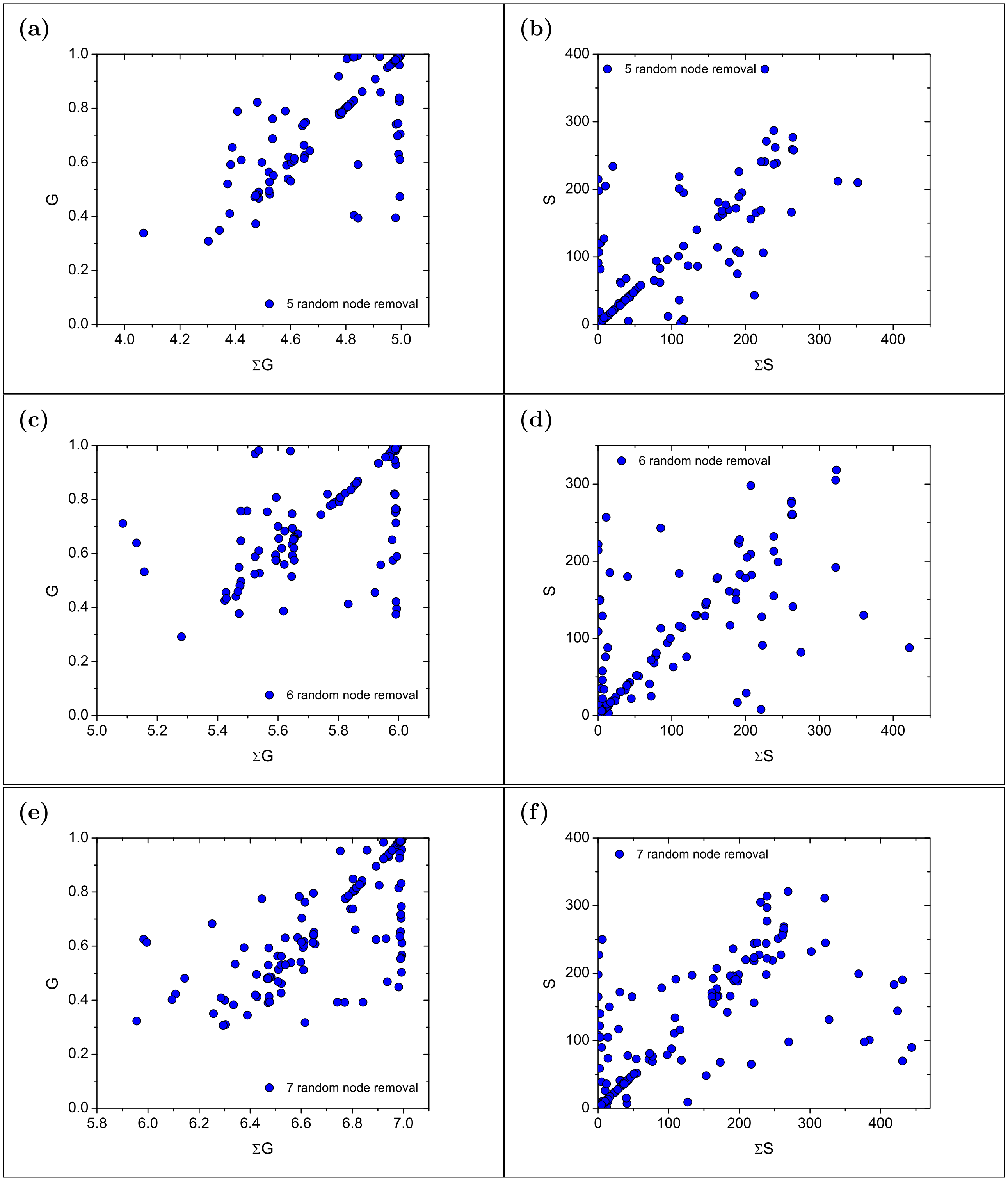

Figure S25: Correlation analysis of multi-node attacks and the sum of individual damage each initiator node induces in N-stable UCTE network.

S.10.1 Outlier removal

To determine accurately which data points are outliers in order to remove them from our analysis, we calculate the prediction intervals and confidence intervals of our data. The confidence interval Fig. S27(blue) reveals the degree of uncertainty associated with a sample statistic, and is calculated from the observations. Prediction intervals or prediction bands Fig. S27(green) establish the interval

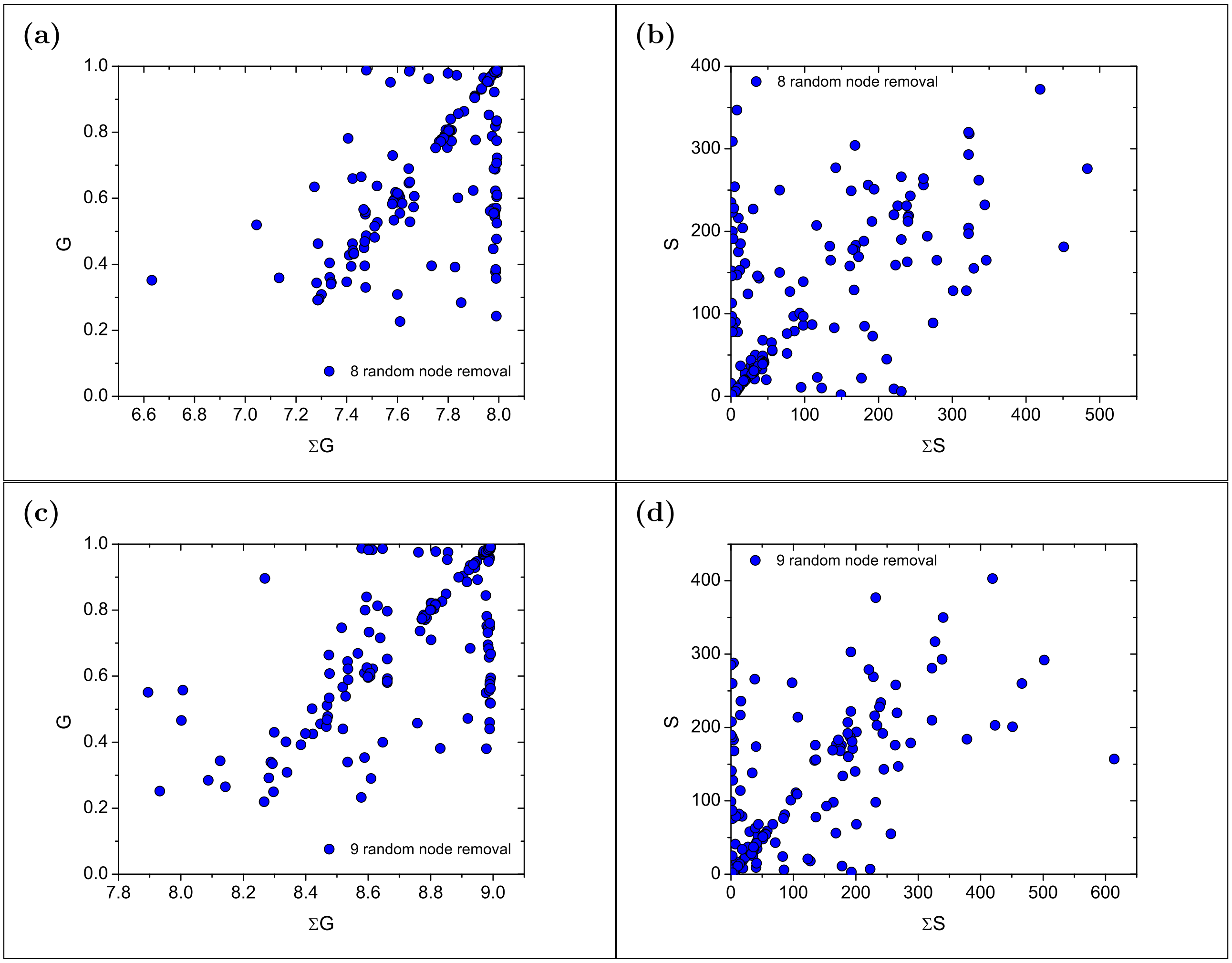

Figure S26: Correlation analysis of multi-node attacks and the sum of individual damage each initiator node induces in N-stable UCTE network.

in which future observations will fall with a certain probability. For our correlation analysis we calculate in confidence interval and prediction bands based on our data, as seen in Fig. S27 insets. Next, we remove all data points that fall outside the prediction bands, and calculate the Pearson correlation coefficient.

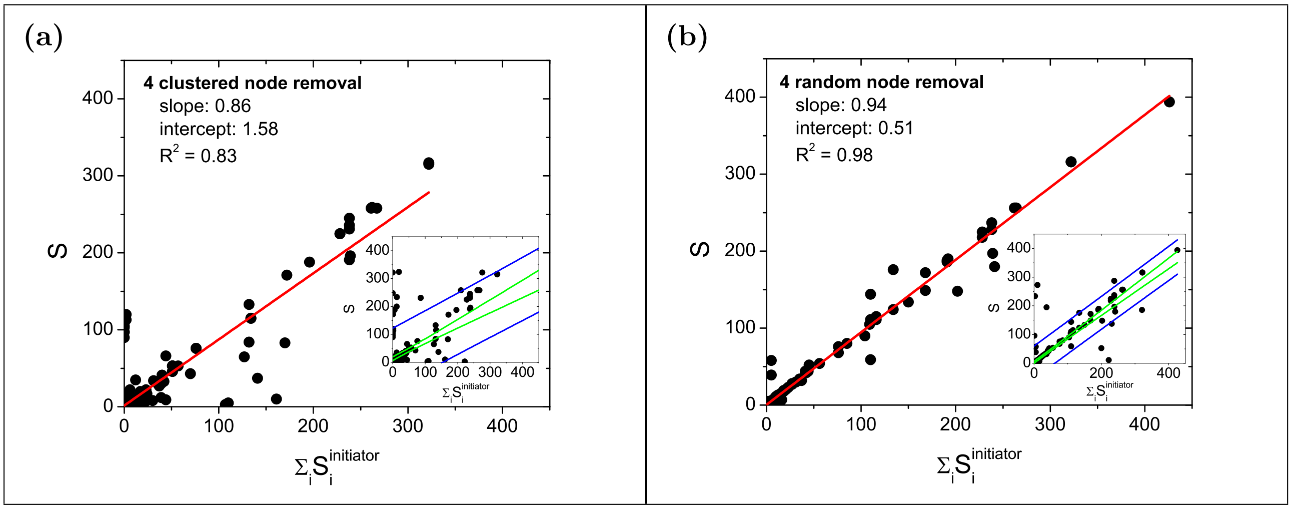

Figure S27: Correlation analysis of failures triggered by the removal of a set of 4 nodes. $\alpha = 0.5927$ (relative tolerance); 300 repetitions of four-random-node removals from the entire network.

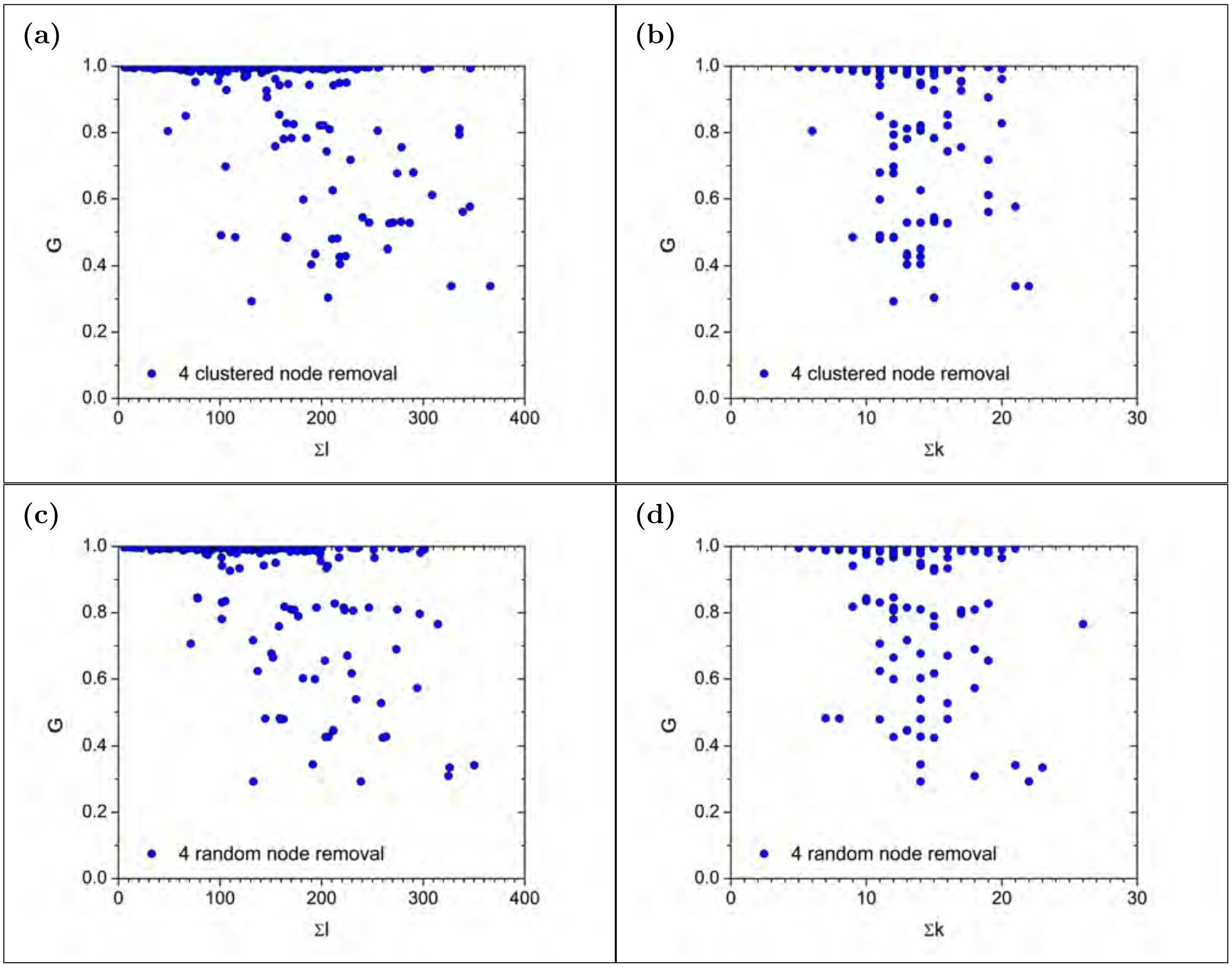

Figure S28: Comparison of four-clustered and four-random-node removals. Subfigures (a) and (b) present the size of the surviving giant component for the four-clustered-node removals as the function of the total node degrees $\sum k$ of the initiator nodes, as well as the total load $\sum \ell$ of initiator nodes. All simulations are performed on UCTE network with $\alpha = 0.60$.

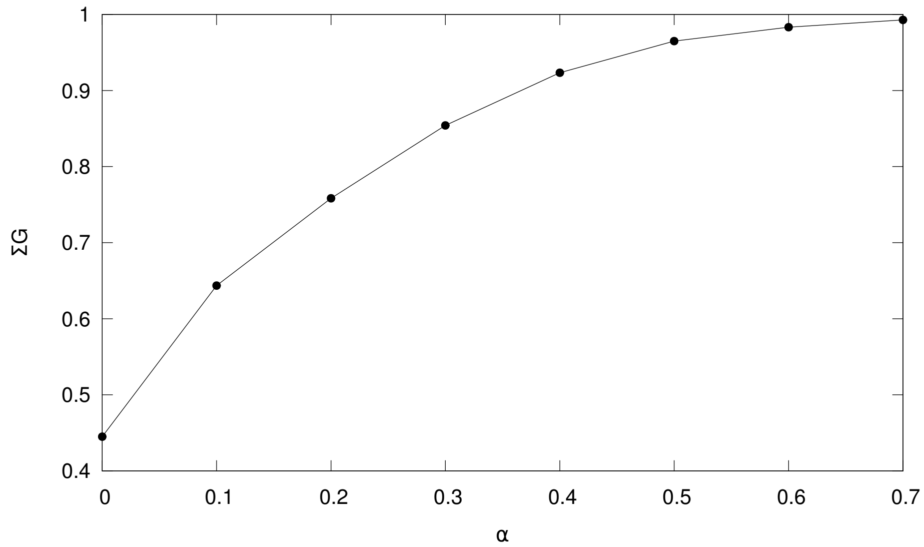

Figure S29: Average G vs α of four-random-node removals from the UCTE network.

References

[1] Asztalos A., Sreenivasan S., Szymanski B.K., Korniss G. Distributed flow optimization and cascading effects in weighted complex networks. *Eur. Phys. J. B* **85**: 288 (2012)

[2] Korniss G., Huang R., Sreenivasan S., Szymanski B.K. Optimizing synchronization, flow and robustness in weighted complex networks. In *Handbook of Optimization in Complex Networks*, edited by Thai MT, Pardalos P, Springer Optimization and Its Applications Vol. 58, Part 1. New York: Springer. 61–96 (2012).

[3] Newman M.E.J. A measure of betweenness centrality based on random walks. *Social Networks* **27**: 39–54 (2005).

[4] Brandes U., Fleischer D. (2005) Centrality measures based on current flow. In: Diekert V, Durand B, editors, Lecture Notes in Computer Science, Springer, NY, volume 3404. 533–544.

[5] Ercsey-Ravasz M., Toroczkai Z. Centrality scaling in large networks. *Phys. Rev. Lett.* **105**: 038701 (2010).

[6] Korniss G., Hastings M. B., Bassler K. E., Berryman M. J., Kozma B., et al. Scaling in small-world resistor networks. *Phys. Lett. A* **350**: 324–330 (2006).

[7] Hernandez V., Roman J.E., Vidal V. SLEPc: A scalable and exible toolkit for the solution of eigenvalue problems. *ACM Trans Math Software* **31**: 351–362 (2005).

[8] Bernstein A., Bienstock D., Hay D., Uzunoglu M., and Zussman G. Power grid vulnerability to geographically correlated failures – analysis and control implications. Technical Report Technical Report No. 2011-05-06, Nov. 2011, Columbia University, Electrical Engineering; https://arxiv.org/abs/1206.1099 (Accessed 2017 February 23).

[9] Verma T., Ellens W., Kooij R. E. Context-independent centrality measures underestimate the vulnerability of power grids. *International Journal of Critical Infrastructures 7* **11** (1), 62-81 (2015).

[10] Pahwa S., Scoglio C., Scala A. Abruptness of Cascade Failures in Power Grids. *Sci. Rep.* **4**: 3694 (2014).

[11] Rahnamay-Naeini M., Wang Z., Ghani N., Mammoli A., Hayat M.M. Stochastic Analysis of Cascading-Failure Dynamics in Power Grids. *IEEE Transactions on Power Systems* **29**(4):1767-1779 (2014).

[12] Zimmerman R., Murillo-Sánchez C., Thomas R. Matpower: Steady-state operations, planning, and analysis tools for power systems research and education. *IEEE Transactions on Power Systems* **26**: 12–19 (2011).

[13] Updated UCTE data (2013) Available: `http://www.powerworld.com/bialek` Accessed 2013 Nov 15.

[14] Motter A. E., Lai Y. C. Cascade-based attacks on complex networks. *Phys. Rev. E* **66**: 065102(R) (2002).

[15] Halu, A., Scala, A., Khiyami, A., and González, M.C. Data-driven modeling of solar-powered urban microgrids. *Science Advance* **2**, e1500700 (2016).

[16] Soltan, S., Mazauric, D., and Zussman, G. Analysis of Failures in Power Grids. *IEEE Transactions on Control of Network Systems* (published online, 2016); https://doi.org/10.1109/TCNS.2015.2498464 (Accessed January 9, 2017).

[17] Korkali M., Veneman J. G., Tivnan B. F. and Hines P. D. H. Reducing Cascading Failure Risk by Increasing Infrastructure Network Interdependency. http://arxiv.org/abs/1410.6836 (2014).

[18] Clauset A., Shalizi C. R., and Newman M. E. J. Power-Law Distributions in Empirical Data. *SIAM Rev.*, **51**(4), 661–703 (2009).

[19] Edwards A. M., Phillips R. A., Watkins N. W., Freeman M. P., Murphy E. J., Afanasyev V., Buldyrev S. V., da Luz M. G. E., Raposo E. P., Stanley H. E., & Viswanathan G. M. Revisiting Lévy flight search patterns of wandering albatrosses, bumblebees and deer. *Nature* **449**, 1044-1048 (2007).
