# Supplementary figures and images for: Limits of Predictability of Cascading Overload Failures in Spatially-Embedded Networks with Distributed Flows
Source: Sci Rep. 2017 Sep 15;7:11729. doi: 10.1038/s41598-017-11765-1 (PMC5601003; doi:10.1038/s41598-017-11765-1)

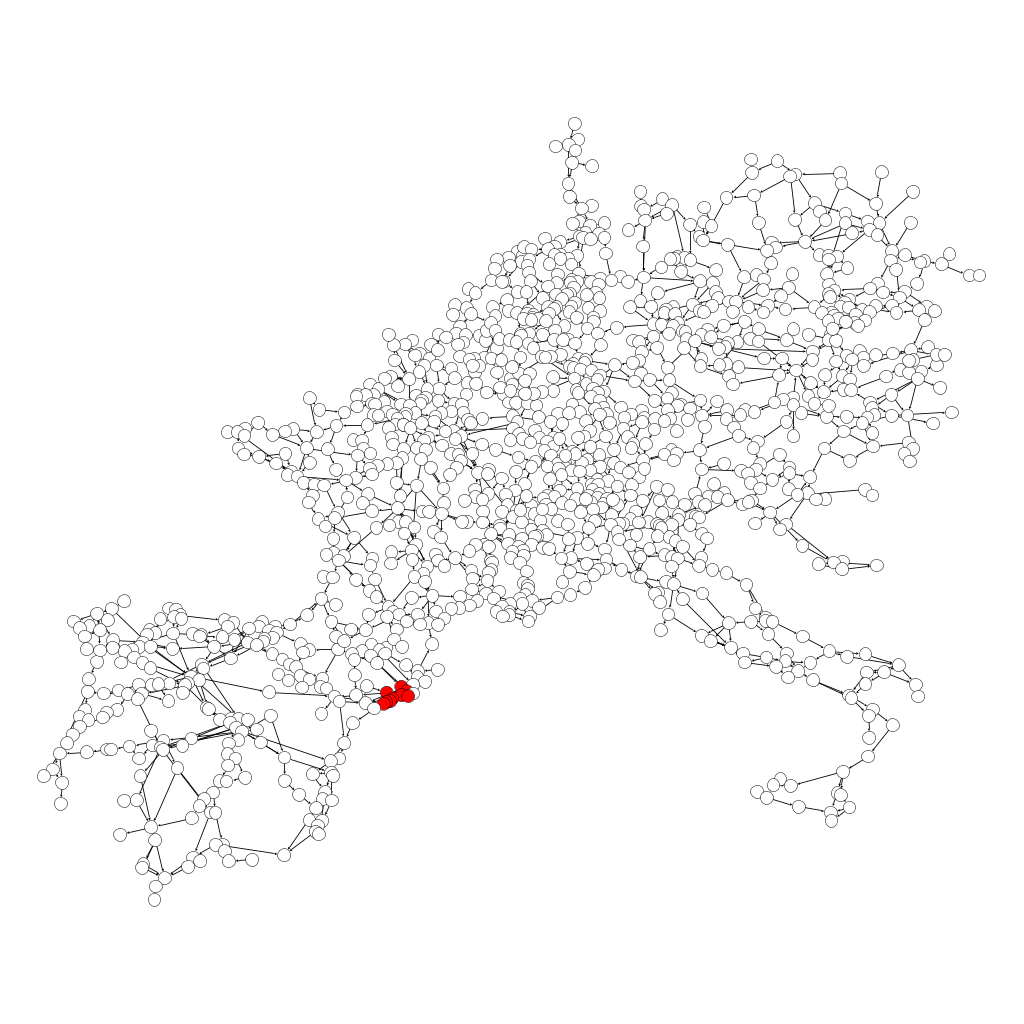

Supplement: Supplementary file 2 — 1r-0.1 [file 41598_2017_11765_MOESM2_ESM.gif]

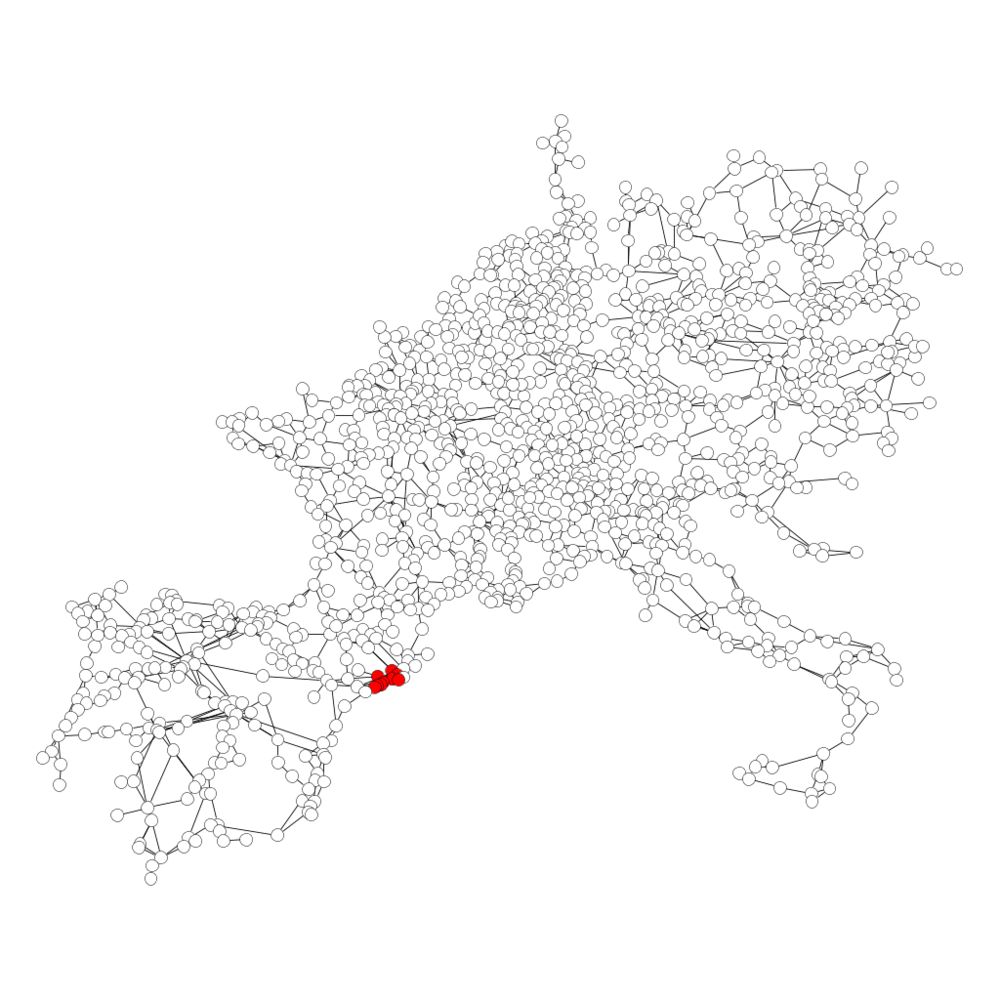

Supplement: Supplementary file 3 — 1r-0.15 [file 41598_2017_11765_MOESM3_ESM.gif]

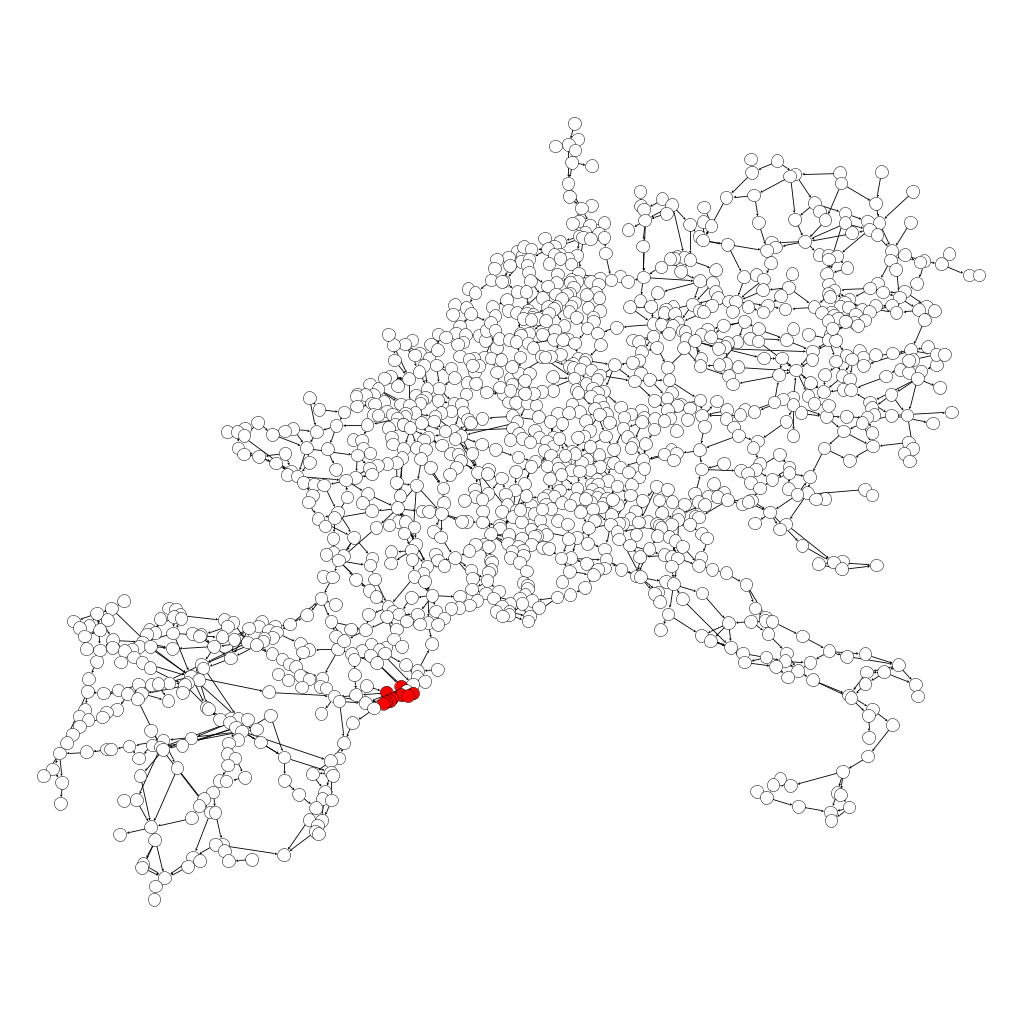

Supplement: Supplementary file 5 — 2r scenario 1 [file 41598_2017_11765_MOESM5_ESM.gif]

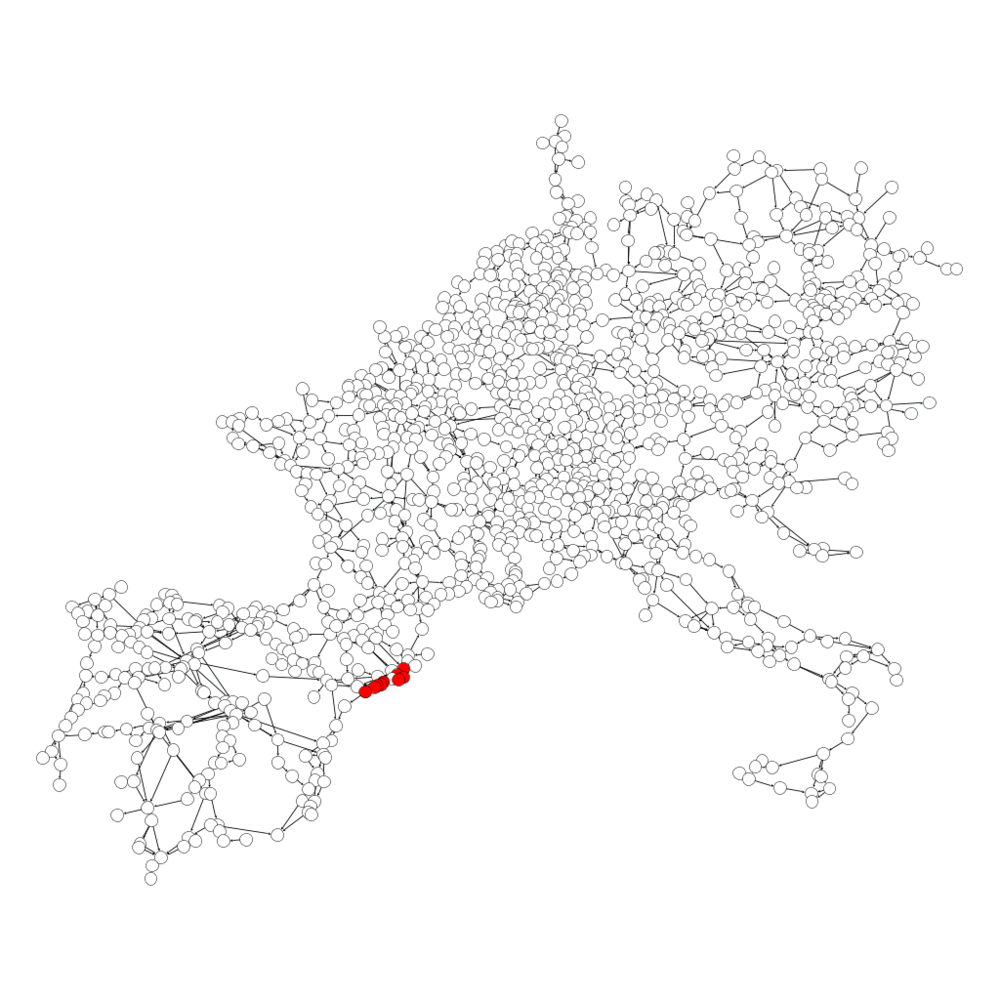

Supplement: Supplementary file 6 — 2r scenario 2 [file 41598_2017_11765_MOESM6_ESM.gif]

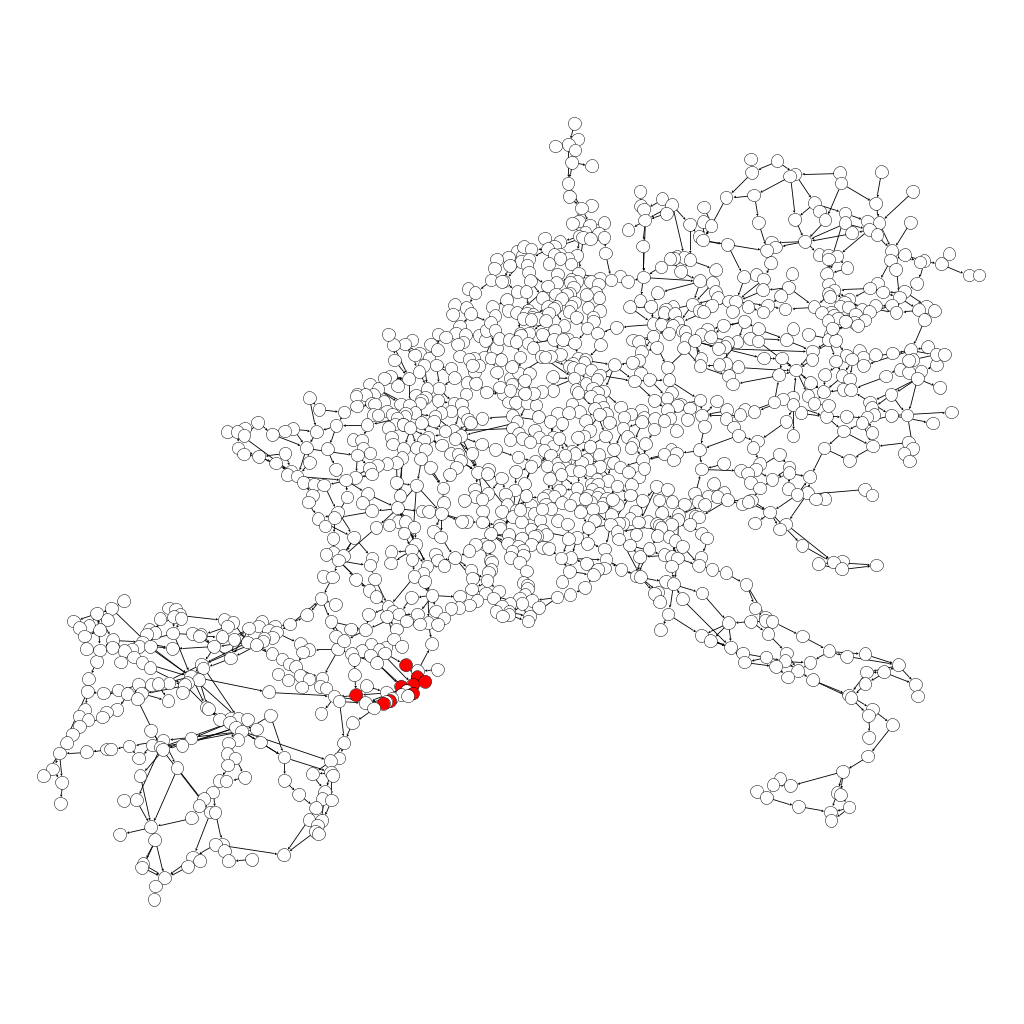

Supplement: Supplementary file 7 — 3r scenario 1 [file 41598_2017_11765_MOESM7_ESM.gif]

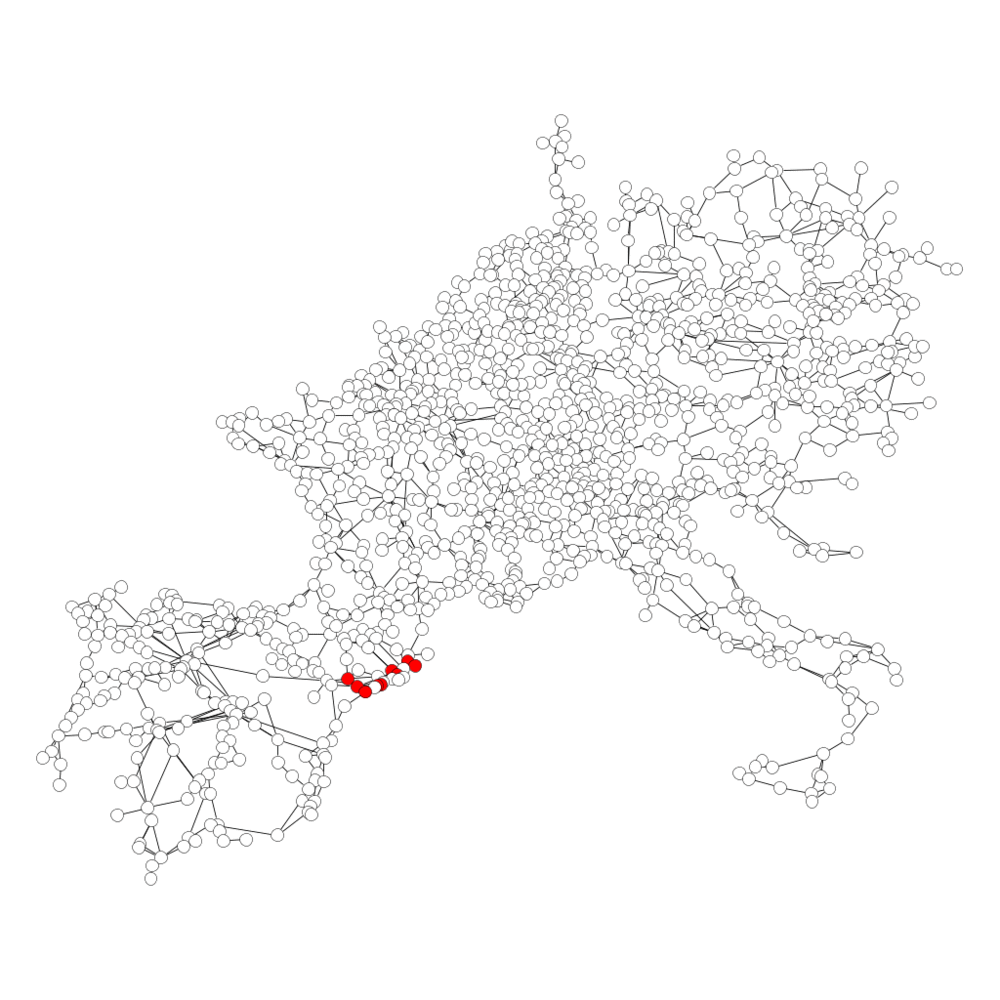

Supplement: Supplementary file 8 — 3r scenario 2 [file 41598_2017_11765_MOESM8_ESM.gif]

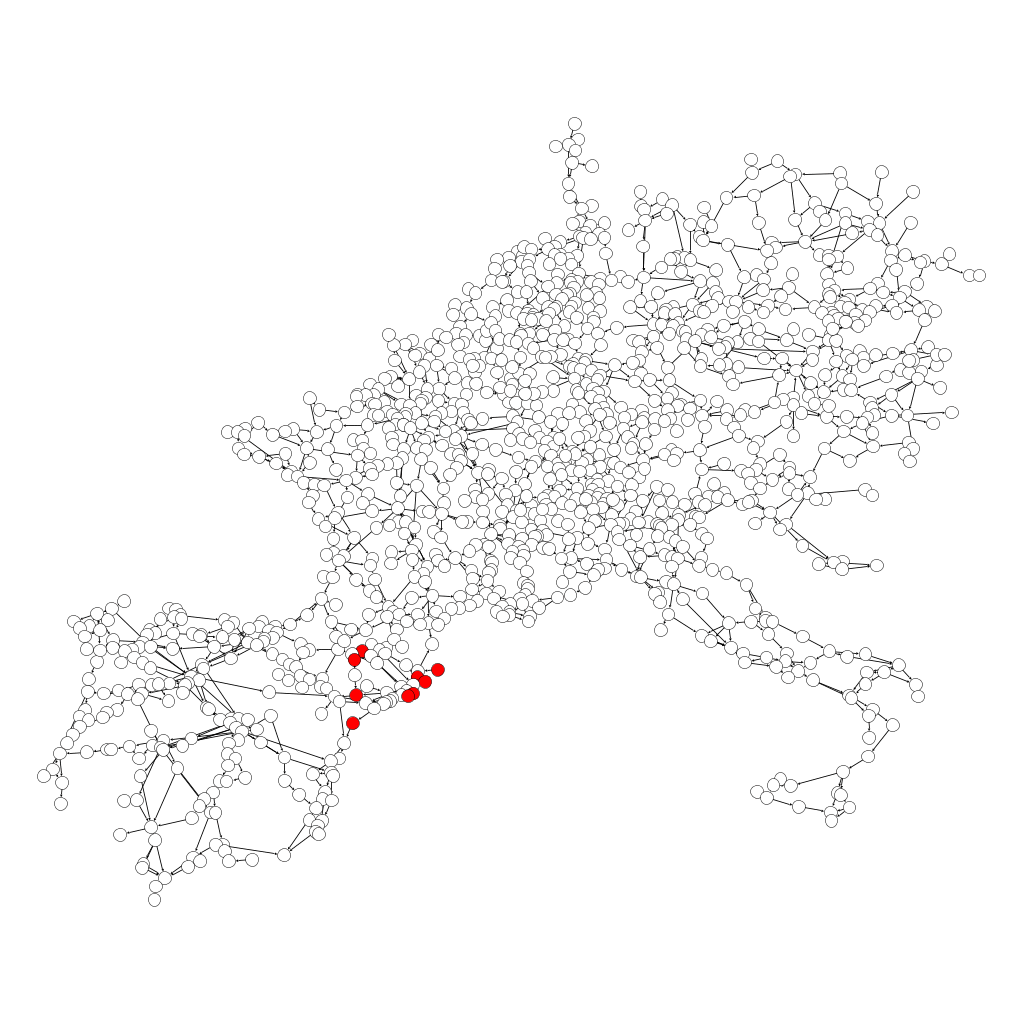

Supplement: Supplementary file 9 — 5r scenario 1 [file 41598_2017_11765_MOESM9_ESM.gif]

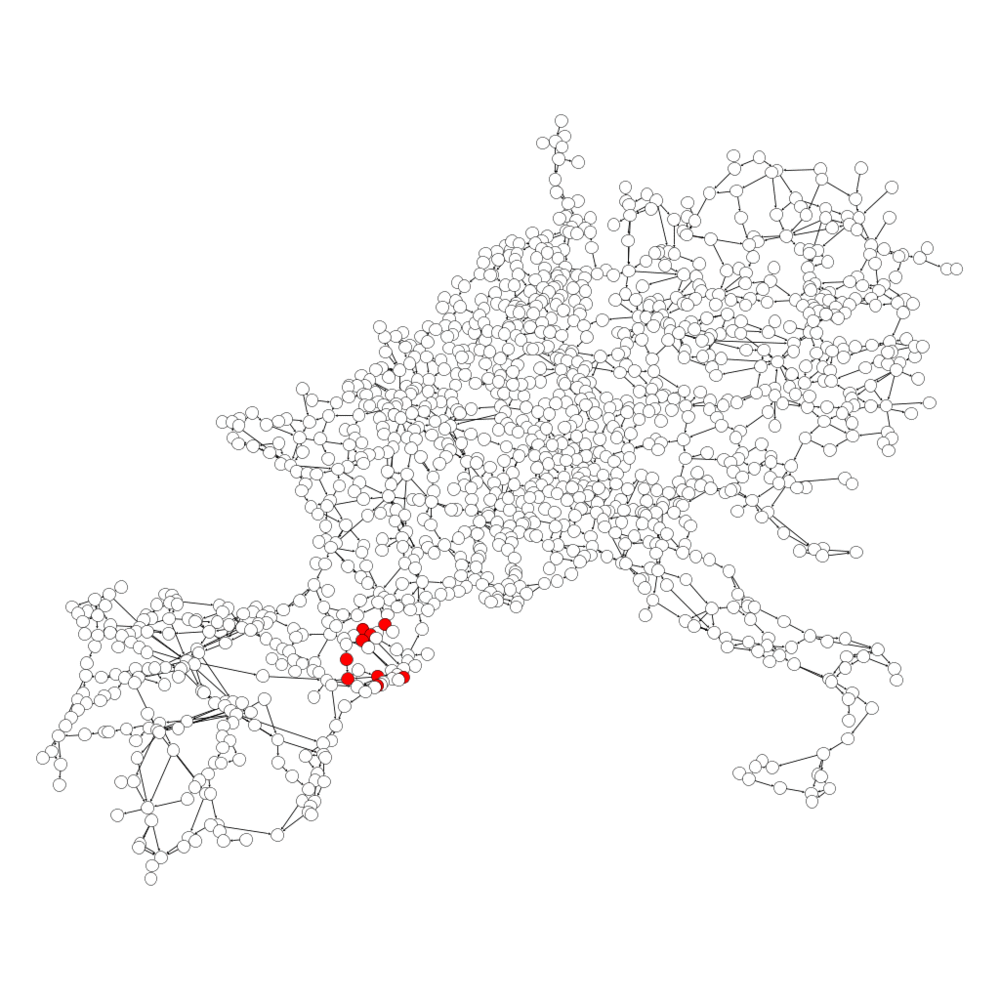

Supplement: Supplementary file 10 — 5r scenario 2 [file 41598_2017_11765_MOESM10_ESM.gif]

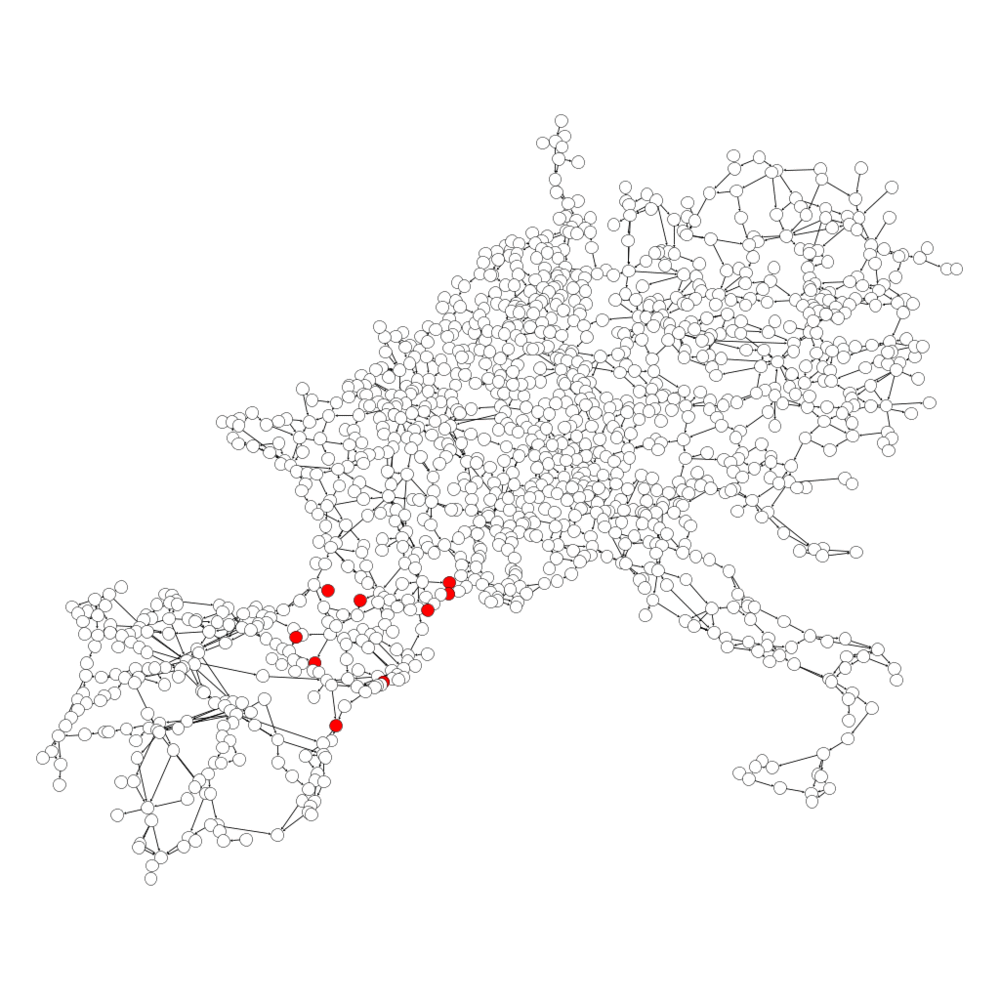

Supplement: Supplementary file 11 — 10r scenario 1 [file 41598_2017_11765_MOESM11_ESM.gif]

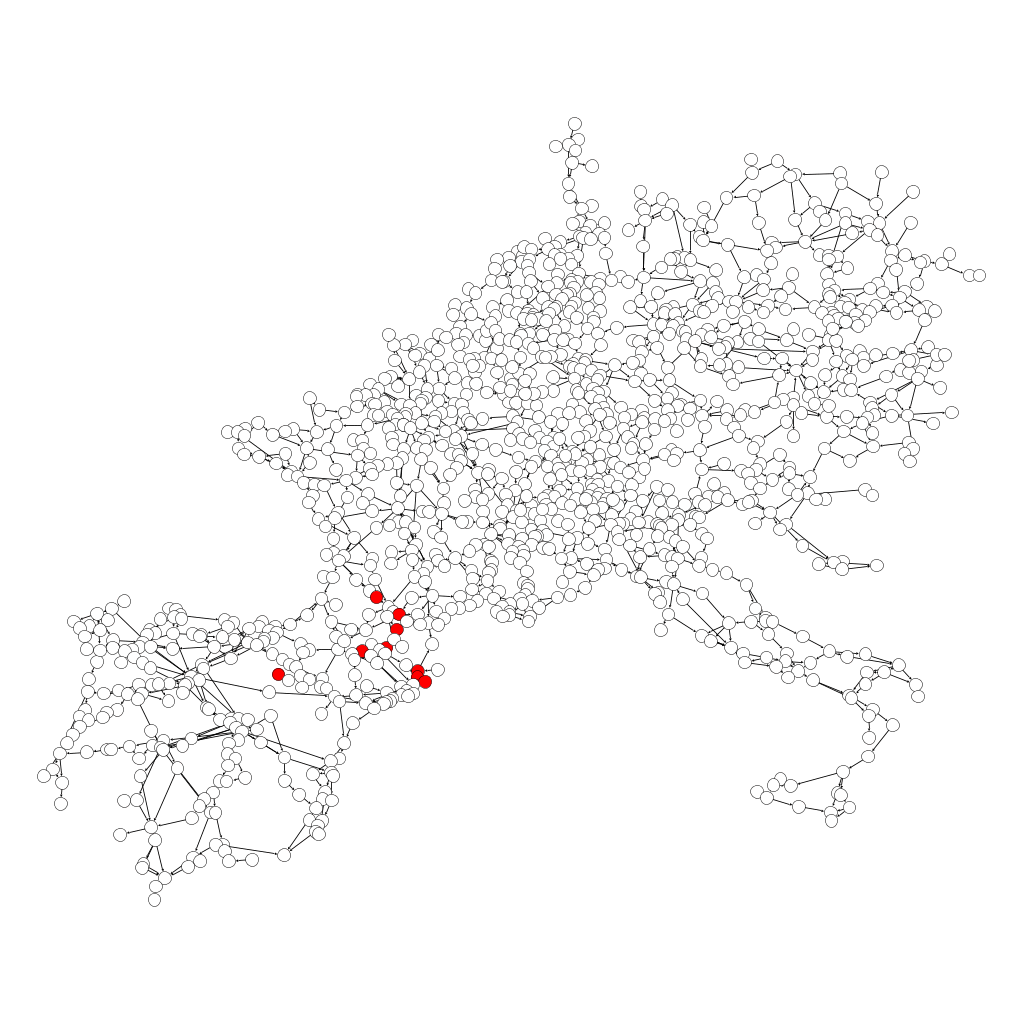

Supplement: Supplementary file 12 — 10r scenario 2 [file 41598_2017_11765_MOESM12_ESM.gif]

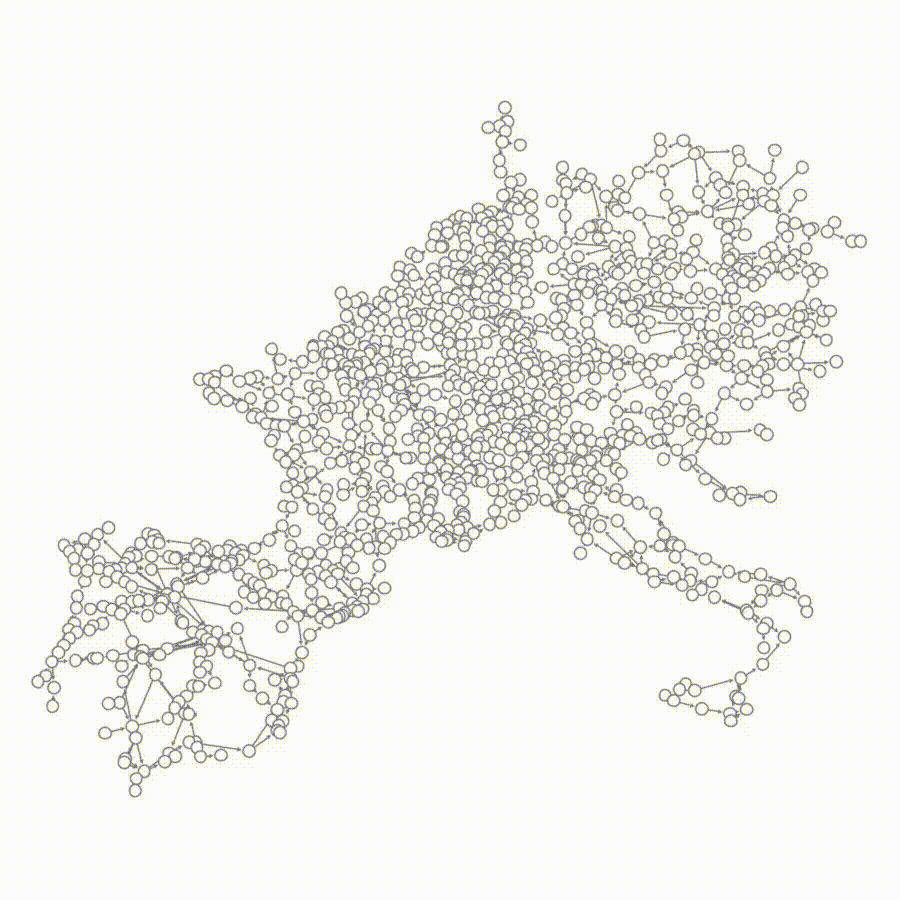

Supplement: Supplementary file 13 — stochastic_alpha0.45_BEST [file 41598_2017_11765_MOESM13_ESM.gif]

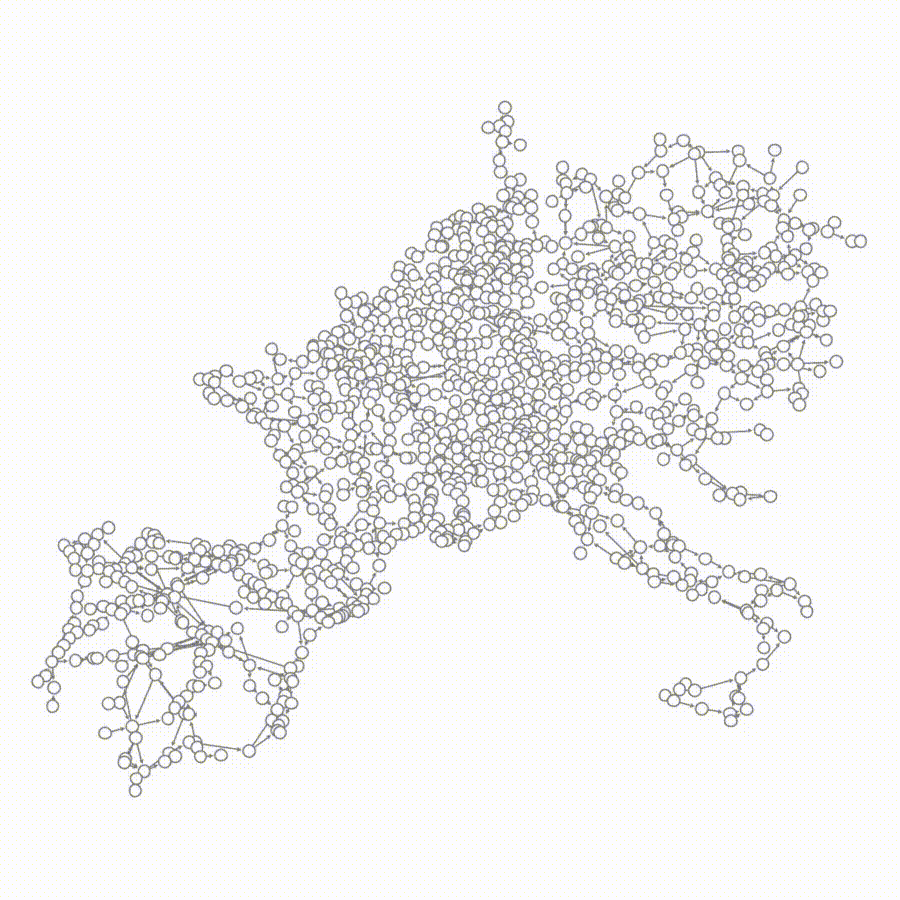

Supplement: Supplementary file 14 — stochastic_alpha0.45_WORST [file 41598_2017_11765_MOESM14_ESM.gif]
